# Complex molecular profile of DNA repair genes in epithelial ovarian carcinoma patients with different sensitivity to platinum-based therapy

**DOI:** 10.3389/fonc.2022.1016958

**Published:** 2022-12-02

**Authors:** Karolina Seborova, Viktor Hlavac, Petr Holy, Sunniva S. Bjørklund, Thomas Fleischer, Lukas Rob, Martin Hruda, Jiri Bouda, Marcela Mrhalova, Mohammad Moufaq Khatar Al Obeed Allah, Pavel Vodicka, Ondrej Fiala, Pavel Soucek, Vessela N. Kristensen, Ludmila Vodickova, Radka Vaclavikova

**Affiliations:** ^1^ Toxicogenomics Unit, National Institute of Public Health in Prague, Prague, Czechia; ^2^ Biomedical Center, Faculty of Medicine in Pilsen, Charles University, Pilsen, Czechia; ^3^ Third Faculty of Medicine, Charles University, Prague, Czechia; ^4^ Department of Medical Genetics, Institute of Clinical Medicine, Faculty of Medicine, University of Oslo, Oslo, Norway; ^5^ Department of Cancer Genetics, Institute for Cancer Research, Oslo University Hospital, The Norwegian Radium Hospital, Oslo, Norway; ^6^ Department of Gynecology and Obstetrics, Third Faculty of Medicine and University Hospital Kralovske Vinohrady, Prague, Czechia; ^7^ Department of Gynecology and Obstetrics, University Hospital in Pilsen, Charles University, Pilsen, Czechia; ^8^ Department of Pathology and Molecular Medicine, Motol University Hospital, Second Faculty of Medicine, Charles University, Prague, Czechia; ^9^ Institute of Experimental Medicine, Czech Academy of Sciences, Prague, Czechia; ^10^ Institute of Biology and Medical Genetics, First Faculty of Medicine, Charles University, Prague, Czechia; ^11^ Department of Oncology and Radiotherapeutics, Faculty of Medicine in Pilsen and University Hospital, Charles University, Pilsen, Czechia; ^12^ Laboratory of Cancer Treatment and Tissue Regeneration, Biomedical Center, Faculty of Medicine in Pilsen, Charles University, Pilsen, Czechia

**Keywords:** ovarian carcinoma, DNA repair genes, resistance, transcriptome, methylome, whole exome sequencing, biomarkers, treatment response

## Abstract

Epithelial ovarian carcinoma (EOC) is known for high mortality due to diagnosis at advanced stages and frequent therapy resistance. Previous findings suggested that the DNA repair system is involved in the therapeutic response of cancer patients and DNA repair genes are promising targets for novel therapies. This study aimed to address complex inter-relations among gene expression levels, methylation profiles, and somatic mutations in DNA repair genes and EOC prognosis and therapy resistance status. We found significant associations of *DUT* expression with the presence of peritoneal metastases in EOC patients. The high-grade serous EOC subtype was enriched with *TP53* mutations compared to other subtypes. Furthermore, somatic mutations in *XPC* and *PRKDC* were significantly associated with worse overall survival of EOC patients, and higher *FAAP20* expression in platinum-resistant than platinum-sensitive patients was observed. We found higher methylation of *RAD50* in platinum-resistant than in platinum-sensitive patients. Somatic mutations in *BRCA1* and *RAD9A* were significantly associated with higher *RBBP8* methylation in platinum-sensitive compared to platinum-resistant EOC patients. In conclusion, we discovered associations of several candidate genes from the DNA repair pathway with the prognosis and platinum resistance status of EOC patients, which deserve further validation as potential predictive biomarkers.

## Introduction

Epithelial ovarian cancer (EOC), one of the most lethal gynecological malignancies, is the eighth leading cause of death among women (1). Over 80% of diagnosed cases of EOC are histologically classified as high-grade serous carcinoma (HGSC) with an aggressive phenotype associated with high mortality (2, 3). The disease is usually diagnosed in advanced stages (FIGO III or IV) when the 5-year survival rate reaches approximately 30% (4–6).

The standard treatment of EOC combines cytoreductive surgery with chemotherapy regimens using platinum derivatives and paclitaxel (7). Recently, new therapeutic approaches have been introduced to the therapy of advanced-stage EOC, e.g., poly (ADP-ribose) polymerase inhibitors (PARPi) represented by olaparib, rucaparib, niraparib, or anti-angiogenic agents represented by bevacizumab (8, 9). PAPRis have been approved by FDA and EMA for EOC patients in the following indications – *BRCA1/2* gene mutation and those with homologous recombination deficiency (HRD). PARPis are mainly used in platinum-sensitive advanced EOC as maintenance therapy (10, 11).

DNA damage response and related DNA repair pathway alterations are important for cancer development, including EOC. Germline mutations in DNA repair genes are predictive for hereditary types of cancer, especially *BRCA1/2* in breast cancer (12) or deleterious mutations in *RAD51C/RAD51D/BRCA1* in ovarian cancer (13–15). Variants in several DNA repair genes, e.g., *BRIP1, RAD50, RAD51C/D, BARD1, CHEK2, MRE11A, PALB2*, and *ATM* are associated with a higher risk of EOC (9). The majority of HGSC cases (96%) harbor *TP53* mutations, which are associated with defective homologous recombination repair through *BRCA1/2* gene mutation(s) (16). Besides homologous recombination repair (*BRCA1/2* and *RAD51C/D*) (15, 17, 18) other repair pathways also seem to be affected, e.g., non-homologous end-joining repair (*XRCC4*) (19), mismatch repair (*MSH2/6, MLH1*, and *PMS2*) (20, 21), base excision repair (*XRCC1*) (22), nucleotide excision repair (*ERCC1*) (23), and direct repair (*MGMT*) (24).

Except for the association of DNA repair genes with the risk of EOC development, these genes are promising potential therapeutic targets and biomarkers for the prediction of therapeutic response. Patients with mutations in *BRCA1/2* respond well to platinum-based chemotherapy and PARPi as proven by many studies (25–28). Among key DNA mismatch repair (MMR) genes, higher gene expression of *MSH6, MLH1*, and *PMS2* is associated with better overall survival of EOC patients treated with platinum-based chemotherapy (21). Concerning methylation, *MSH2* was significantly hypermethylated in resistant EOC patients (20). Promoter methylation of the other MMR gene, *MLH1* was associated with acquired resistance to platinum-based chemotherapy in EOC patients (29). Resistance to cisplatin was observed in EOC patients with higher expression of base excision repair (BER) pathway core genes such as *XRCC1* (22) and carriage of polymorphisms in *ERCC1* (rs11615 and rs3212986) from the nucleotide excision repair (NER) pathway (23). Among other BER genes, *MUTYH* was recently suggested for inclusion in the panel of chemotherapy-responsive genes in EOC (30). To the best of our knowledge, complex analysis of intersections between DNA repair gene expression, methylation profiles, and somatic genetic variability largely missing in current literature.

In the present study, 178 DNA repair genes were selected for complex investigation comprising the vast majority of the human DNA repair system. Analysis of gene expression profile by RNA sequencing (RNAseq) approach, microarray methylation profile and somatic genetic variability by the whole exome sequencing (WES) technology was performed. The aims of the study were (i) addressing the associations between the examined expression profiles and therapy response of EOC patients, especially differences between patients with platinum-resistant and platinum-sensitive status after adjuvant chemotherapy and (ii) identification of intersections between gene expression profiles, genetic variability, and methylation profiles of significant DNA repair genes. The ultimate aim of this study was to reveal potential biomarkers of EOC prognosis and development of resistance.

## Methods

### Patients’ samples

The tissue samples for the present study were obtained from 73 patients with EOC diagnosis from University Hospital Kralovske Vinohrady (Prague, Czech Republic) and University Hospital in Pilsen (Pilsen, Czech Republic). The tissue specimens collected during surgery were histopathologically verified, immediately fresh frozen, and stored at -80°C until further processing.

The following personal and clinico-pathological data were retrieved from patient´s medical records: age at diagnosis, tumor grade, FIGO stage, histological type, adjuvant chemotherapy regimens, presence of peritoneal metastases and residuum after surgery. All assembled clinico-pathological data for patients enrolled in the present study are summarized in **Table 1**. An independent ovarian cancer cohort was used for validation of results of the present study – The Cancer Genome Atlas Ovarian Serous Adenocarcinoma (TCGA-OV; gene expression data – level 1, DNA methylation data – level 3, downloaded from https://tcga-data.nci.nih.gov) cohort described in **Table S1** in the **Supplementary material** (31).

**Table 1:** Detailed clinical characteristics of EOC patients enrolled in the gene expression profile, methylation profile and WES profile analyses.

Characteristics	Expression profile^+^	Methylation profile^+^	Genetic profile^+^
	N (%)	N (%)	N (%)
**Number of patients**	**60**	**73**	**52**
**Age** (mean, years ± SD)	60.1 ± 9.7	59.2 ± 9.7	59.7 ± 9.4
**Stage**			
I	3 (5)	3 (4.1)	2 (3.8)
II	3 (5)	3 (4.1)	3 (5.8)
III	49 (81.7)	62 (84.9)	42 (80.8)
IV	4 (6.6)	4 (5.4)	4 (7.7)
Not available	1 (1.7)	1 (1.5)	1 (1.9)
**EOC type**			
HGSC	42 (70)	54 (73.9)	36 (69.3)
Others	17 (28.3)	18 (24.6)	15 (28.8)
Not available	1 (1.7)	1 (1.5)	1 (1.9)
**Histological Grade**			
G1	5 (8.3)	5 (6.8)	5 (9.7)
G2	9 (15)	10 (13.7)	6 (11.5)
G3	46 (76.7)	58 (79.5)	41 (78.8)
**Peritoneal Metastases**			
Present	4 (6.7)	4 (5.5)	4 (7.7)
Absent	54 (90)	65 (89)	47 (90.4)
Not available	2 (3.3)	4 (5.5)	1 (1.9)
**Residuum after surgery**			
Present	33 (55)	42 (57.5)	28 (53.8)
Absent	26 (43.3)	31 (41)	24 (46.2)
Not available	1 (1.7)	1 (1.5)	0 (0)
**Regimen of chemotherapy**			
Taxane with platinum derivatives^1^	57 (95)	66 (90.4)	50 (96.2)
Other regimens^2^	3 (5)	6 (8.2)	2 (3.8)
Unknown	0 (0)	1 (1.4)	0 (0)
**Platinum resistance status**			
Platinum-sensitive	37 (61.6)	43 (58.9)	32 (61.5)
Platinum-resistant	23 (38.4)	30 (41.1)	20 (34.5)
**Platinum free interval (PFI)**			
All patients (mean ± SD; months)	25 ± 25	22 ± 24	24 ± 25
Sensitive patients (mean ± SD; months)	38 ± 24	34 ± 24	37 ± 24
Resistant patients (mean ± SD; months)	4 ± 3	5 ± 3	4 ± 3

^1^ Regimen based on combination of paclitaxel with carboplatin/cisplatin.

^2^ Other regimen containing platinum monotherapy and combination of carboplatin +/- paclitaxel with Avastin.

^+^ All types of analyses were performed in 52 EOC patients. For gene expression (N=60) and methylation profiles (N=73), more samples of sufficient quality were available.

Informed consent was obtained from all participants included in the study. All procedures performed in this study followed the ethical standards of the Institutional Review Boards of the National Institute of Public Health in Prague, University Hospital Kralovske Vinohrady and University Hospital in Pilsen, and the 1964 Helsinki declaration and its later amendments or comparable ethical standards. The experimental protocol of this study was also approved by the Institutional Review Boards of the National Institute of Public Health in Prague, University Hospital Kralovske Vinohrady, and University Hospital in Pilsen. This article does not contain any research using animals.

### Isolation of nucleic acids and quantity/quality determination

Tumor tissue samples were ground to powder by mortar and pestle under liquid nitrogen. Total RNA and DNA were isolated by AllPrep DNA/RNA/Protein Mini Kit (Qiagen, Hilden, Germany) following manufacturer´s protocol. RNA and DNA were quantified using the Quant-iT RiboGreen RNA Assay Kit and the Quant-iT PicoGreen dsDNA Assay Kit, respectively (both Invitrogen, Waltham, MA, USA), on the plate reader Infinite M200 (Tecan Group ltd., Switzerland). Quality of isolated RNA was estimated by determination of RNA integrity number (RIN) on Bioanalyzer 2100 (Agilent Technologies Inc., Santa Clara, CA, USA) using the RNA 6000 Nano kit (Agilent Technologies Inc.). Purity of RNA and DNA samples was verified by Nanodrop 2000 (ThermoFisher Scientific, Waltham, MA, USA) and calculation of ratios A260/A280 and A260/A230.

### RNA sequencing library preparation and sequencing

For RNA sequencing analysis, total RNA from 60 patients with RIN > 6.4 (mean RIN 8.5, range 5.4 – 10) was used. Library preparation was performed using 500ng input of total RNA using the QuantSeq 3´mRNA-Seq Library Prep FWD for Illumina kit (Lexogen, Vienna, Austria) according to manufacturer’s protocol. Quality of libraries was checked by Bioanalyzer 2100 using High Sensitivity DNA kit (Agilent Technologies Inc.) and quantity was measured by qPCR, using KAPA Library Quantification Kit for Illumina Platforms (F.Hoffmann-La Roche AG, Basel, Switzerland). The equimolar pool of prepared libraries was sequenced on the NextSeq 500 platform (Illumina Inc., San Diego, CA, USA) in one run of the 75 cycle High-Output kit, targeting > 6M reads per sample.

### High-throughput DNA methylation profiling

Estimation of DNA methylation profile was performed in a set of 73 EOC patients. At first, bisulfite conversion of 500 ng DNA was done using EZ DNA MethylationTM Kit (Zymo Research, Irvine, CA, USA) according to the manufacturer´s manual. Estimation of genome-wide DNA methylation level for more than 850,000 methylation sites across the genome were done by Infinium MethylationEPIC BeadChip microarray (Illumina Inc.) according to the manufacturer´s recommendations. Microarray was scanned by iSCAN System (Illumina Inc.).

### Whole exome sequencing library preparation and sequencing

DNA libraries were prepared from fresh frozen tumor tissues and matched blood samples of 52 patients (N=52). 100 ng of DNA was used as input. SureSelect XT Low Input for Illumina and Enzymatic Fragmentation Kit (Agilent Technologies Inc.) were used for the preparation of libraries according to the manufacturer´s protocol. In each capture reaction, eight libraries were pooled equimolarly based on qPCR quantification (Kapa Library Quantification Kit, F.Hoffmann-La Roche AG). Hybridization was performed using SureSelect Human All Exon V7 (Agilent Technologies Inc.) according to standard protocol. Tumor and blood libraries were pooled in ratio 9:1 before sequencing. Sequencing was performed on the NovaSeq 6000 system (Illumina Inc.) using S4 chemistry (version 1.5) with 2 x 150 cycle setup.

### Data analysis

#### mRNA expression analysis

Quality of raw RNA sequencing data was performed by the *fastp* package (32). The GENCODE v35 (GRCh38.p13) reference transcriptome was used for gene annotation (33). Abundance of protein-coding genes was estimated by the pseudoalignment approach using *kallisto* (34). For gene differential expression analysis, the *EdgeR* package was implemented (35). Genes with P-values<0.05 were considered differentially expressed.

#### Methylation analysis

**Figure 1:**
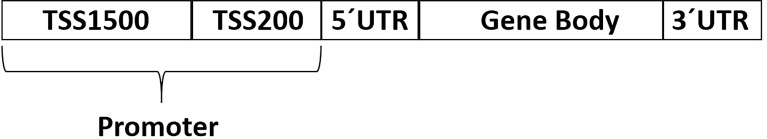
Gene regions selected for methylation analysis using Infinium MethylationEPIC BeadChip microarrays of the cassette of 178 DNA repair genes in EOC patients (N = 73).

Quality control and initial normalization was performed by the SWAN approach in the *minfi* package as described previously (36–38). Raw data were converted to β values (for graphical illustration) and M values (for statistical analysis) (39–41). Analysis of data from methylation arrays included filtering of probes with annotated single nucleotide polymorphism (SNP), which were filtered out based on the list published by Pidsley et al. (42). For analysis of gene regions the probes were then collapsed into specific gene regions based on the manifest for the microarray – TSS200 (CpG between TSS (transcription start site) and 200bp upstream and TSS itself), TSS1500 (CpG between TSS and 1500 – 200bp upstream), 5´UTR (CpG in 5´UTR), 1stExon (CpG in the first exon), gene body (CpG in other exons or introns), 3´UTR (CpG in 3´UTR region) as shown in **Figure 1**. Promoter region was defined by the combination of CpGs in TSS200 and TSS1500. We focused on whole gene, TSS200, TSS1500, and promoter methylation profiles. Differential methylation analysis was done using the *limma* package (43). Due to the problematic analysis of whole gene DNA methylation profile we decide for simplification in this case and use median of M values for a specific region/gene for statistical analysis

#### WES analysis

For the DNA sequencing analysis, raw data were first demultiplexed by the *bcl2fastq* software while separating unique molecular barcodes (UMIs). Quality control of FASTQ files was performed using the *FastQC* 11.9 software (44). Trimming was performed by the *AGenT Trimmer 2.0.3* (Agilent Technologies) software. Alignment of both read pairs to the GRCh38 reference genome was done by the *Burrows-Wheeler aligner* (45) and deduplication by the *AGenT Locatit 2.0.5* software (Agilent Technologies), utilizing UMIs. Quality control and manipulation of BAM files was performed using the *Qualimap 2.2* (46) and *Samtools 1.13* (47) packages, respectively. The packages *vcftools 1.16* (48) and *bcftools* 1.13 (47) were used for VCF file manipulation. Base recalibration and somatic variant calling were conducted using the *Genome Analysis Toolkit 4* (GATK4) (49). The variant caller *Mutect2* utilized tumor-normal paired samples from the same patients, and the gnomAD v2 (50) database as a germline variability resource. The raw calls were filtered, as well as all previous and subsequent steps were performed, according to the GATK Best Practices (51, 52). Annotation of variants was done using Funcotator (GATK) (53). Comparisons of mutation rates and general somatic variant analyses were performed using the R package *maftools 2.10* (54). All open-source bioinformatics tools were obtained from Bioconda v2.8 (55) or Bioconductor v3.13 (56).

#### Statistical analyses with clinical data

For analysis of associations of gene expression levels with clinical characteristics of patients, normalized data from RNA sequencing in the format of transcripts per million (tpm) were used. Statistical analyses were performed using non-parametric tests (the Mann-Whitney, the Kruskal-Wallis, or the Spearmanś rank correlation test) using the SPSS software v16.0 (IBM, Armonk, NY, USA) or GraphPad Prism v4.0 (GrapPad Software Inc, San Diego, CA, USA). Type I error in single gene expression analyses was controlled by the false discovery rate (FDR) test according to Benjamini and Hochberg (57) for analysis of gene expression with clinico-pathological data, or by the Bonferroni correction in the case of differential expression analysis in the *EdgeR* package (35).

Analysis of associations between β values representing methylation status and clinico-pathological data was done using SPSS software v16.0 with the same statistical tests as for gene expression levels. The FDR was handled as above.

Analysis of associations between genetic profiles and clinico-pathological data was carried by the Pearson´s chi-square test. Patients were marked as mutated in a specific gene when having any mutation of moderate or high impact (missense, frameshift, nonsense, nonstop, splice site) in that gene, otherwise they were counted as non-mutated.

The survival functions were computed by the Kaplan–Meier method. Evaluation of EOC patient’s survival (OS, in months) was based on the interval from the date of surgery to the date of death or last follow up. Platinum resistance status was estimated as the interval elapsed between the date of the last dose of platinum-based chemotherapy and the date of relapse/progression, death or last follow up (based on PFI – platinum free interval, in months) (58). Cut-offs defined by quartiles were tested and the “optimal cut-off” was defined as the highest statistical significance by the log-rank test. Patients were divided by the median value of expression/methylation for a specific gene or by the presence/absence of a specific somatic mutation.

## Results

### Patient´s characteristics

Gene expression profiles of 178 DNA repair genes were analyzed in a cohort of 60 EOC patients, where RNA samples were available in appropriate quality. DNA samples of EOC patients (N=73) were used for the methylation study. WES analysis was successfully performed in 52 DNA tumor EOC samples, which were available in sufficient quantities. Relevant clinical data of EOC patients included in the study are shown in **Table 1**. The mean of the patients´ age at diagnosis was ~ 60 years. The majority of patients´ tumor samples were histologically classified as HGCS (72%) at Stage III (82%) and Grade 3 (79%). Peritoneal metastases were found in four patients (7%). Chemotherapy regimens combined mostly paclitaxel with carboplatin (63%) or paclitaxel with carboplatin and cisplatin (28%). Patients with PFI < 6 months and 6 – 12 months (N=30) were defined as platinum-resistant and patients with PFI > 12 months (N=43) were assigned as platinum-sensitive. The mean PFI was ~ 4.3 months for platinum-resistant and ~ 36.3 months for platinum-sensitive patients participating in the study.

Characteristics of patients from the TCGA database used for validation of our results are shown in **Table S1** in the **Supplementary Material**.

### Expression profile of DNA repair genes

The gene expression profile of the examined panel of 178 DNA repair genes (see **Table S2** in the **Supplementary Material**) was estimated using RNA sequencing. Continuous normalized levels of target genes in EOC patients were evaluated for their associations with available clinical data. As shown in **Table 2** significant associations of *HUS1, PMS2, POLH, RECQL5, RPA1*, and *XAB2* gene expression with the patients´ age were observed. The presence of peritoneal metastases was significantly associated with higher expression of *DUT* and *FANCI*, even that only four EOC patients had metastases. The presence of residuum after surgery was associated with higher *PALB2* and *TDG* gene expression. EOC patients with advanced tumor grade (Grade 3) had higher *PCNA* expression. Aggressive HGSC type of EOC was associated with higher levels of *PCNA, ERCC2, ALKBH3, TOBP1*, and inversely with low levels of *LIG3, FAN1, MSH3*, and *XPC* genes. Among all observed relationships with prognostic factors, only that of *DUT* expression with the presence of peritoneal metastases passed the FDR correction (P=0.0003, **Table 2**) (**Figure S1** in the **Supplementary Material**).

**Table 2:** The significant associations of DNA repair gene expression profiles with clinical data of EOC patients (N = 60, only significant results with P ≤ 0.01 are shown).

Gene	Age	pM	Residuum	Grade ^a^	EOC type ^b^
*HUS1*	P=0.008	NS	NS	NS	NS
*PMS2*	P=0.01	NS	NS	NS	NS
*POLH*	P=0.009	NS	NS	NS	NS
*RECQL5*	P=0.009	NS	NS	NS	P=0.001
*RPA1*	P=0.005	NS	NS	NS	NS
*XAB2*	P=0.005	NS	NS	NS	NS
***DUT***	NS	**P=0.0003**	NS	NS	NS
*FANCI*	NS	P=0.01	NS	NS	NS
*PALB2*	NS	NS	P=0.002	NS	NS
*TDG*	NS	NS	P=0.007	NS	NS
*ERCC2*	NS	NS	NS	NS	P=0.01
*PCNA*	NS	NS	NS	P=0.001	P=0.001
*ALKBH3*	NS	NS	NS	NS	P=0.006
*FAN1*	NS	NS	NS	NS	P=0.003
*MSH3*	NS	NS	NS	NS	P=0.008
*TOBP1*	NS	NS	NS	NS	P=0.002
*XPC*	NS	NS	NS	NS	P=0.006
*LIG3*	NS	NS	NS	NS	P=0.004
*RECQL4*	NS	NS	NS	NS	P=0.01
*PMS1*	NS	NS	NS	NS	NS

a Patients divided into two groups: Group 1 (Grade 1 and Grade 2), Group 2 (Grade 3).

b Patients divided into two groups: Group 1 (HGSC type), Group 2 (Others type).

Significant result after FDR correction is displayed in bold. NS, not significant.

**Figure 2:**
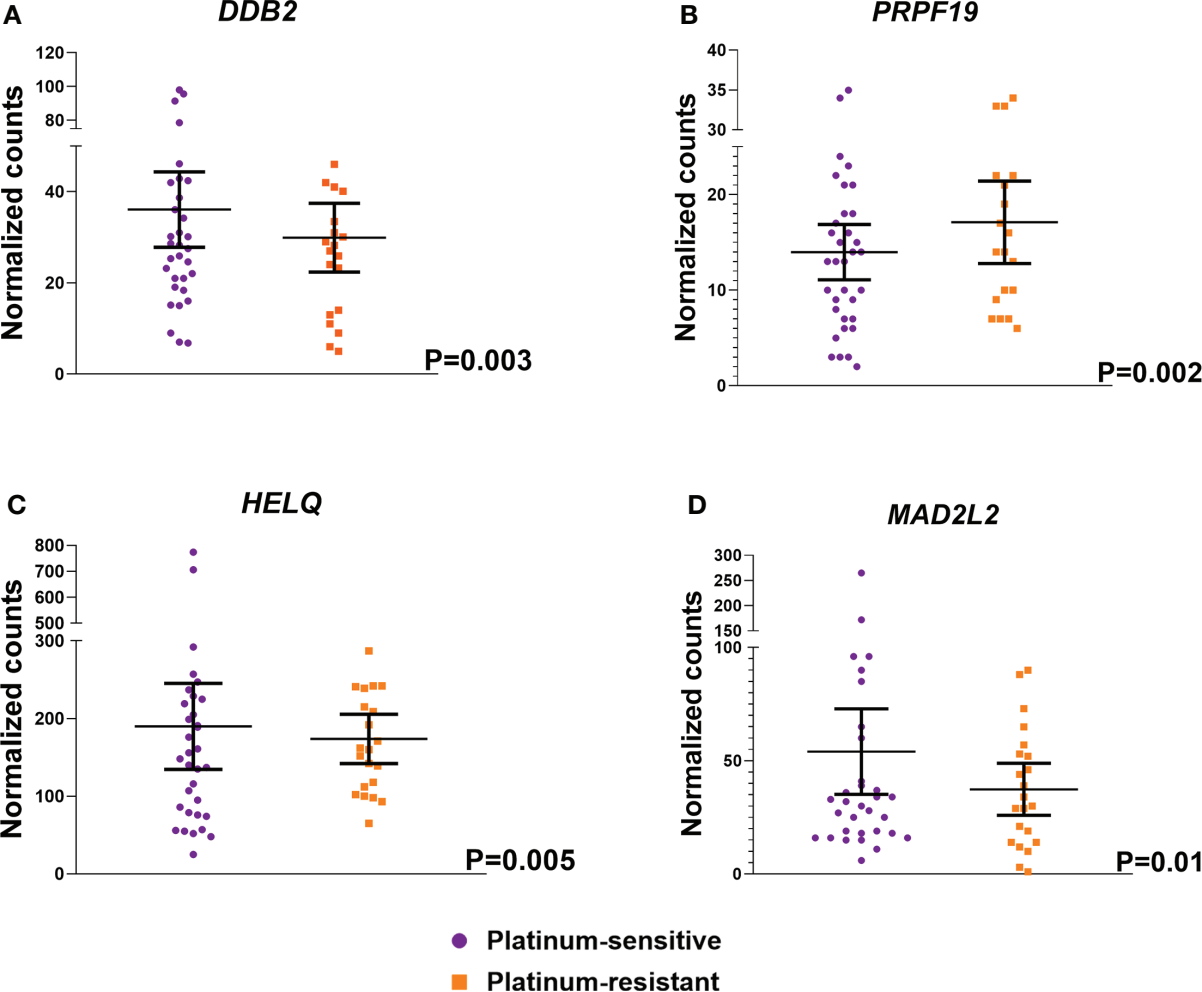
Differentially expressed DNA repair genes based on the response of EOC patients to platinum based chemotherapy. Analysis of differential expression between platinum-sensitive patients and the platinum-resistant ones showed four differentially expressed genes – **(A)** *DDB2*, **(B)** *PRPF19*, **(C)** *HELQ* and **(D)** *MAD2L2*. EOC patients were divided as platinum-sensitive (N=37, purple color) and platinum-resistant (N=23, orange color) based on the platinum resistance status determined by platinum-free interval (PFI).

Subsequently, differential expression analysis of 178 DNA repair genes was performed in the examined cohort of 60 EOC patients. It was focused mainly on the platinum resistance status of EOC patients based on PFI. Significantly higher expression of *DDB2* (logFC -0.55, P<0.0003), *HELQ* (logFC -0.48, P=0.005), and *MAD2L2* (logFC -0.63, P=0.01) genes was found in platinum-sensitive patients compared to the platinum-resistant ones (**Figure 2**). On the other hand, the expression of *PRPF19* (logFC 0.89, P=0.002) was significantly lower in platinum-sensitive EOC patients. However, none of these associations passed the FDR test for multiple comparisons.

Results from differential expression analysis were evaluated in available RNAseq data from the TCGA database. Only 168 patients from TCGA database were suitable for validation, based on known chemotherapy regimen corresponding with our patients and completed follow-up. The majority of EOC patients with evaluated platinum resistance status in TCGA database are sensitive to the platinum-based chemotherapy (N=137) in comparison to very few platinum-based chemotherapy resistant EOC patients (N=31). In the TCGA dataset, higher expression of *SMUG1* (logFC -0.37, P=0.0017), *FAN1* (logFC -0.45, P=0.008), *DCLRE1C* (logFC -0.29, P=0.01), and *MAD2L2* (logFC 0.34, P=0.034) genes in platinum-resistant patients compared to platinum-sensitive ones was found. However, none of the results from TCGA data passed the FDR test for multiple comparisons. Validation of our findings using TCGA data is limited due to the large differences in available EOC samples (i.e. intra-tumor heterogeneity).

### Methylation profile of DNA repair genes

Methylation profiles of 178 DNA repair genes were estimated in 73 tumor DNA samples of EOC patients. Strong associations of the whole gene methylation profile for *APLF, FAN1, PARP3*, and *POLL* with the patients´ age were found (**Table 3**). The presence of peritoneal metastases was associated with lower methylation of the whole *POLM* gene. High tumor grade was associated with higher methylation of *RAD51C* and *TREX1* and lower methylation of *OBFC2B.* Significant associations of lower methylation profiles of *APEX2, ERCC2, FANCB, RAD51C, HUS1*, and *MSH5* with HGSC subtype and, on the opposite, higher methylation of *MPG* with the HGSC subtype were discovered (**Table 3**). Among all significant results, only the association of *POLL* methylation profile with age passed the FDR test, and we observed a positive correlation (R=0.441) between the higher methylation profile of *POLL* and higher age.

**Table 3:** Associations of whole DNA repair gene methylation profile with clinical data of EOC patients (N=73, only significant result with P ≤ 0.01 are listed).

Gene	Age	pM	Grade ^a^	EOC type ^b^
*APLF*	P=0.009	NS	NS	NS
*FAN1*	P=0.007	NS	NS	NS
*PARP3*	P=0.01	NS	NS	NS
***POLL***	**P=0.0002**	NS	NS	NS
*APEX2*	NS	NS	NS	P=0.004
*ERCC2*	NS	NS	NS	P=0.004
*FANCB*	NS	NS	NS	P=0.001
*RAD51C*	NS	NS	P=0.003	P=0.001
*HUS1*	NS	NS	NS	P=0.008
*MPG*	NS	NS	NS	P=0.007
*MSH5*	NS	NS	NS	P=0.001
*POLM*	NS	P=0.002	NS	NS
*OBFC2B*	NS	NS	P=0.003	NS
*TREX1*	NS	NS	P=0.007	NS

a Patients divided into two groups: Group 1 (Grade 1 + Grade 2), Group 2 (Grade 3).

b Patients divided into two groups: Group 1 (HGSC type), Group 2 (Other types).

Significant result after FDR correction in bold. NS, not significant.

**Figure 3:**
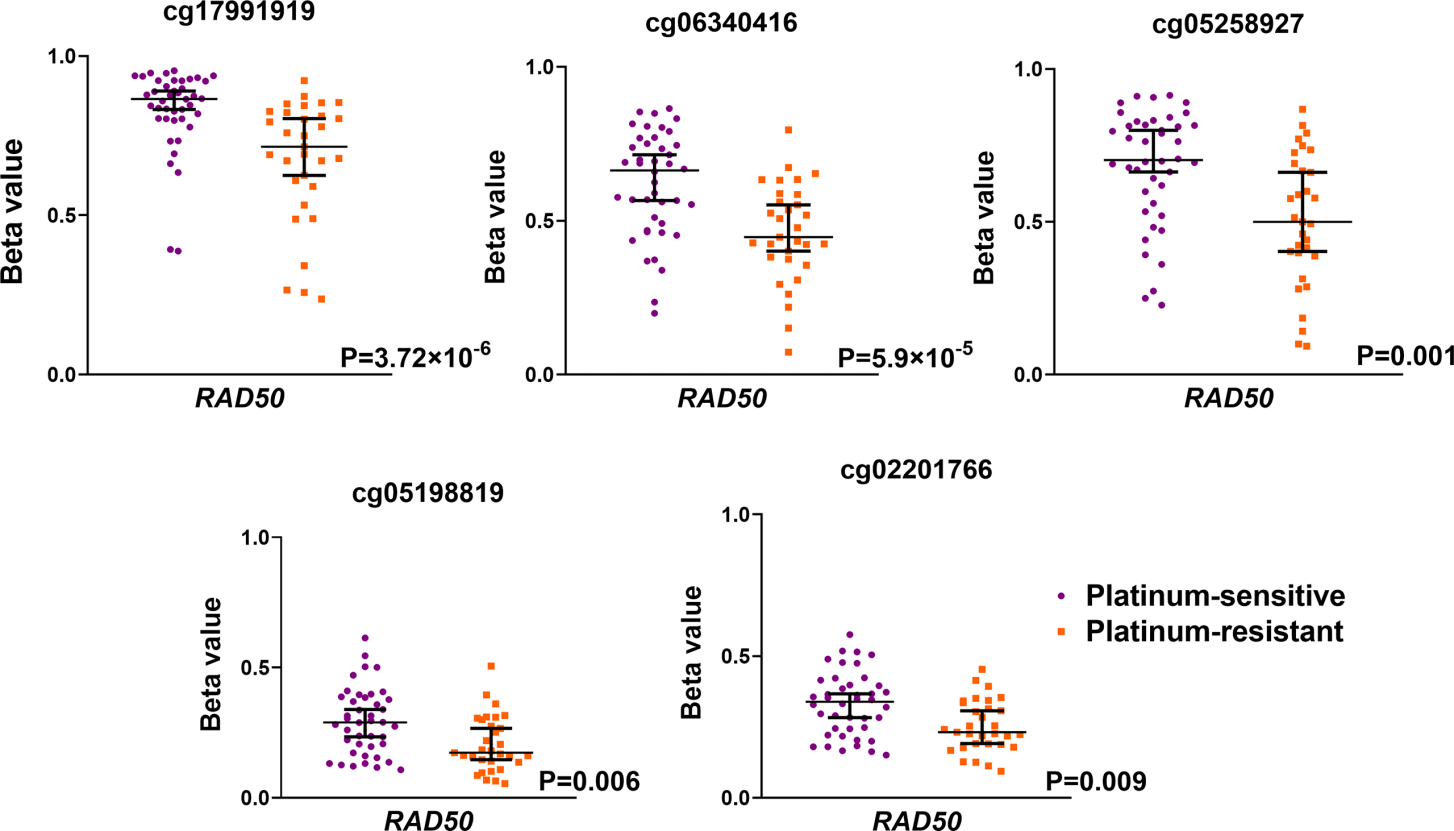
Significantly differentially methylated probes covering *RAD50* gene as estimated using Infinium MethylationEPIC BeadChip microarrays. EOC patients were divided as platinum-sensitive (N = 43, purple color) and platinum-resistant (N = 30, orange color) based on the platinum resistance status determined by platinum-free interval (PFI).

Further, methylome profiles of selected DNA repair genes were compared with platinum resistance status. A summary of probes covering estimated DNA repair genes is shown in **Table S3** in the **Supplementary Material**. In total, 50 differentially methylated probes were identified in comparison of methylome profiles between patients with different platinum resistance status (**Table S4** in the **Supplementary Material**). Among the most significantly differentially methylated probes was the *RAD50* gene as shown in **Figure 3**. A higher methylation profile of *RAD50* gene probes located in the TSS1500 gene region was observed in platinum-sensitive patients compared to platinum-resistant ones. The other significantly differentially methylated probes were found in different gene regions of *FANCD2, GTF2H3, NHEJ1, MBD4*, and *RAD51C* as summarized in **Table S5** in the **Supplementary Material**. On the whole gene level, only one differentially methylated gene was found *– XRCC4*. Results on TSS200, TSS1500, and promoter levels are summarized in **Table S5** in the **Supplementary Material**. Our study is the first type of EOC set of patients defined by platinum resistance status analyzed using the advanced Infinium MethylationEPIC methylation array, covering over 850,000 methylation sites across the genome.

In the TCGA database, data for most of the patients come from the previous version of the Illumina 27K methylation array. Data from the extended 450K Illumina methylation array are available only for 10 EOC patients in the TCGA database.

### Genetic variability of DNA repair genes

Somatic genetic variability of the 178 DNA repair gene panel was analyzed in 52 available EOC patients. Patients with the HGSC subtype had significantly more *TP53* somatic mutations than patients with other subtypes (P=0.001 after FDR). Specific *TP53* somatic mutations are listed in **Table S6** in the **Supplementary Material**. We found 31 different potentially impactful somatic mutations in the *TP53* gene (19 missense, 4 nonsense, 3 splice site, and 5 frameshift deletion mutation type) in 39 EOC patients. Furthermore, having these somatic mutations in *CHEK1, FANCH, MLH3, MMS19, POLD1*, and *RPA2* genes associated with higher stage (Stage III/Stage IV, P=0.007 after FDR).

### Complex analysis of DNA repair genes expression, methylation and genetic variability profile intersections

Individual expression and methylation profiles as well as genetic profiles were compared to each other using available bioinformatics tools. Overlap for comparison of DNA repair genetic variability with gene expression/methylation profiles was 52 EOC patients.

#### Correlation of DNA repair genetic variability and gene expression profiles using expression quantitative trait loci analysis (eQTL)

At first, the effect of the somatic variation profile (variants with potential protein impact) of 178 examined DNA repair genes on gene expression profiles was analyzed. In EOC patients bearing *XPC* somatic mutations (two variants identified - rs750450365 andp.E433K) we found higher expression of *ERCC2* (P=0.003), *RECQL5* (P=0.009), and *FAAP20* (P=0.04) genes and for EOC patients bearing *PRKDC* mutations (three variants identified - p.E3448G, p.Y1243R, and p.L1242fs) we discovered higher *FAAP20* expression, (P=0.03) in all patients (**Table S7** in the **Supplementary Material**).

**Figure 4:**
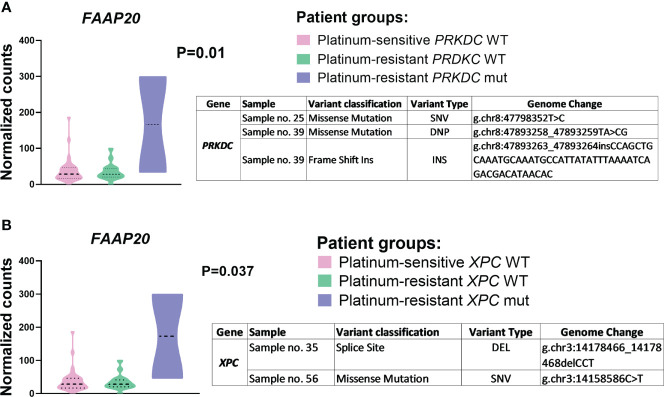
*FAAP20* gene expression compared to **(A)** *PRKDC* and **(B)** *XPC* mutations. Differential expression of *FAAP20* between patient groups – platinum-sensitive (N = 32, pink color), platinum-resistant harboring mutant *PRKDC/XPC* genes (N = 2, purple color) and platinum-resistant harboring wild type *PRKDC/XPC* genes (N = 18, green color). All P-values are declared after adjusting for FDR. Patients were divided based on the platinum resistance status determined by platinum-free interval (PFI). SNV (single nucleotide polymorphism/variant), DEL (deletion), DNP (double nucleotide polymorphism/variant), INS (insertion).

Next, the effect of somatic genetic variability of the DNA repair gene panel on their gene expression in EOC patients divided by the platinum resistance status was investigated. A significantly higher expression of *FAAP20* was found in platinum-resistant EOC patients bearing somatic mutation of X*PC* (P=0.01) or *PRKDC* (P=0.037) (N=2) compared to platinum-resistant patients bearing wild type form of these genes (N=18) (**Figure 4**).

In TCGA database, only one patient bearing *XPC* somatic mutation and six patients bearing *PRKDC* somatic mutations were retrieved. The same trend in eQTL between *FAAP20* and *PRKDC* mutations (six variants identified - p.K2716R, p.G3646Afs*4, p.A2960T, p.V3600L, p.P3972Q, and p.K2220Nfs*18) and platinum resistance status (P=0.038) was confirmed, although this association did not pass the FDR correction (**Figure S2** in the **Supplementary Material**). At present, there are no available data from cohorts of EOC patients large enough for comparison of our results with patients carrying the examined mutation profile.

Overview of the particular *XPC, PRKDC* damaging somatic mutations in our dataset, also in the TCGA database is in the **Table S6** in the **Supplementary Material**.

#### Correlation of DNA repair genes variability and methylation profile using methylation quantitative trait loci analysis (mQTL)

Differential methylation analysis of the examined DNA repair genes revealed a number of significant associations with their genetic variability in EOC patients. Analysis was done over the entire gene regions including whole gene, TSS200, TSS1500, and promoter region. The most important finding was observed for mutations in *BRCA1* (two variants identified - p.L1476fs and g.chr17:43076608delA) and *RAD9A* (two variants identified - p.E184K and p.G24R), which were significantly associated with *RBBP8* methylation levels in all examined regions of the gene (**Table S8** in the **Supplementary Material**).

**Figure 5:**
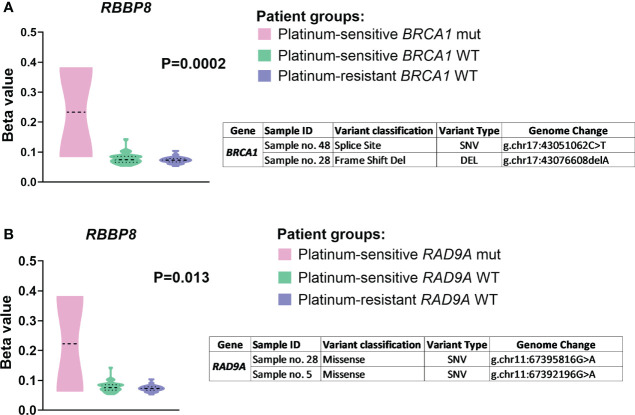
The *RBBP8* methylation profile divided by **(A)** *BRCA1* and **(B)** *RAD9A* somatic mutations. Patients were stratified to groups – platinum-sensitive harboring wild type *BRCA1/RAD9A* genes (N = 30, green color), platinum-resistant harboring wild type *BRCA1/RAD9A* genes (N = 20, purple color), and platinum-sensitive harboring mutant *BRCA1/RAD9A* genes (N = 2, pink color) based on the platinum resistance status determined by platinum-free interval (PFI). Displayed P values are after FDR correction. SNV (single nucleotide polymorphism/variant), DEL (deletion).

The analysis stratified by the platinum resistance status supported these results as platinum-sensitive patients bearing *BRCA1/RAD9A* somatic mutations (N=2) had higher *RBBP8* methylation compared to platinum-sensitive ones bearing *BRCA1/RAD9A* wild type genes (N=30) or platinum-resistant EOC patients (N=20) where no mutations were found in both genes (**Figure 5**).

#### Correlation of methylation and expression profiles of DNA repair genes

In the next step, the median M values for gene, TSS200, TSS1500, or promoter methylation were correlated with normalized data from RNA sequencing by the Spearman rho´s correlation test. A negative correlation of the whole gene methylation level with the expression of *BRCA1, FANCB*, and *MSH2* was revealed (**Table S9** in the **Supplementary Material**). A positive correlation of TSS200 methylation profile with the expression of *ERCC1, MUTYH*, and *PER1* genes and a positive correlation of the TSS1500 methylation profile with the expression of *MUTYH* gene were observed as well. On the other hand, a negative correlation of methylation in the TSS1500 site with the expression of the *FANCG* gene was found. Correlation between methylation in the promoter gene region with expression was observed for *ALKBH2* (positive) and *RAD9A* (negative). However, these results did not pass the FDR correction and they need to be validated in larger cohort of patients.

### Survival analysis

Finally, survival analysis was performed based on our significant results – gene expression (*DDB2, PRPF19, HELQ, MAD2L2*, and *FAAP20*), methylation (*RAD50* and *RBBP8*), and genetic variability (*PRKDC, XPC, BRCA1* and *RAD9A*). No significant association between expression or methylation profiles of the selected genes and overall survival of EOC patients was found, but patients bearing somatic mutations in *XPC* (rs750450365 and p.E433K) or *PRKDC* (p.E3448G, p.Y1243R, and p.L1242fs) had significantly shorter survival (P=0.017 and P=0.037, respectively) (**Figure S3** in the **Supplementary Material**).

Survival analysis focused on combination of our results showed significant difference in overall survival for groups of EOC patients with *BRCA1, RAD9A* wild-type genes in connection with *RAD50* methylation, longer overall survival was found for EOC patients with higher *RAD50* methylation (P=0.011) (**Figure S4A** in the **Supplementary material**). Analysis of somatic mutations of *XPC, PRKDC* with *FAAP20* expression showed shorter overall survival for EOC patients with these mutations and higher *FAAP20* expression (P=0.028) (**Figure S4B** in the **Supplementary material**).

### Biological function of significant changes in gene profiles in relation to the platinum-based treatment

**Figure 6:**
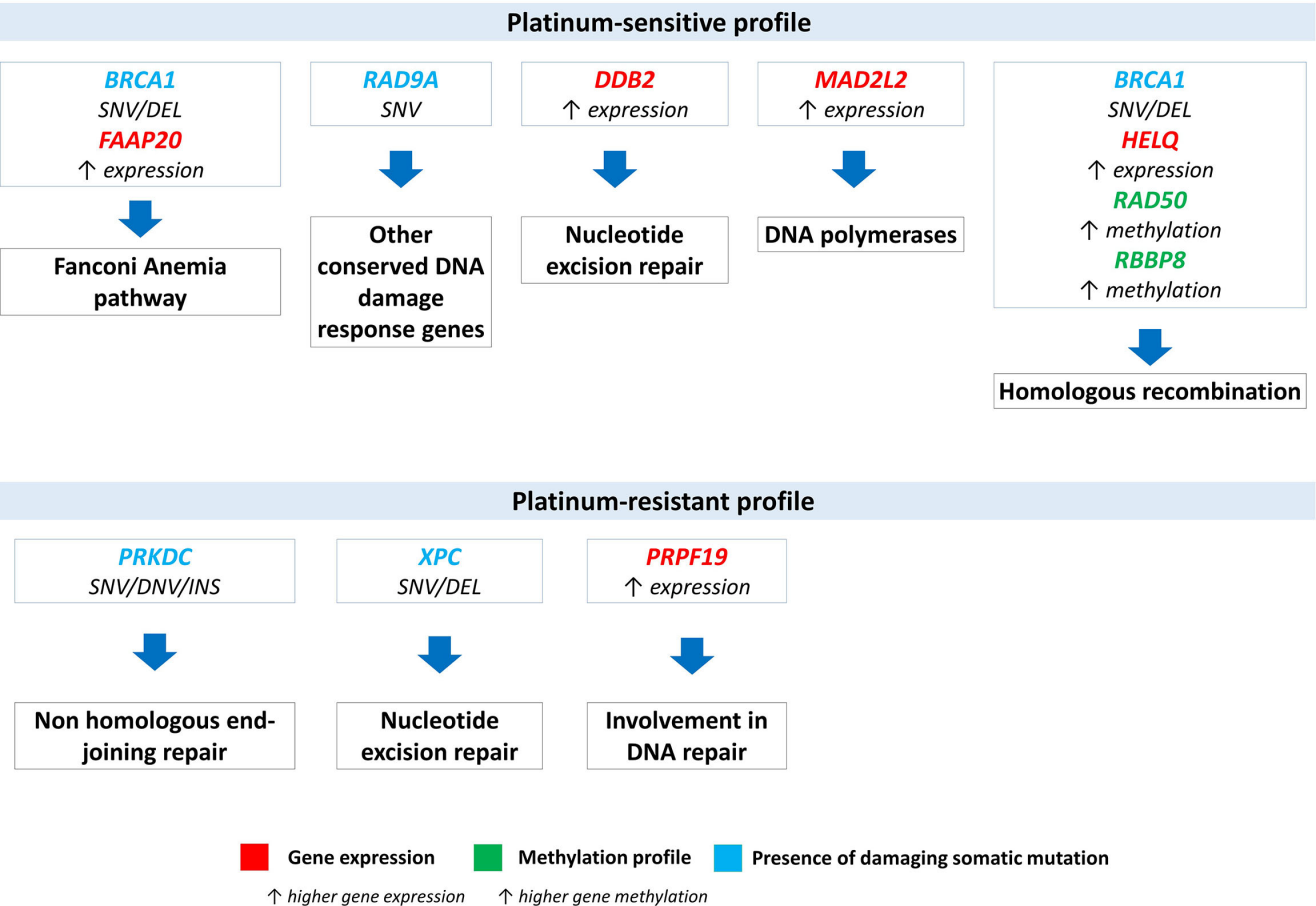
Overview and pathway classification of DNA repair genes with significant deregulation based on platinum-resistant status. Significant expression changes (*DDB2, PRPF19, HELQ, MAD2L2* in red), significant changes in methylation profile (*RAD50*), significant intersections from eQTL analysis (*XPC, PRKDC* and *FAAP20*) and significant intersections from mQTL analysis (*BRCA1, RAD9A* and *RBBP8* in blue). SNV (single nucleotide variant), DEL (deletion), DNV (double nucleotide variant), INS (insertion).

Significantly deregulated genes or genes with changes in methylation and mutation profiles were analyzed in terms of their biological functions. The most significant changes between platinum-sensitive and platinum-resistant EOC patients involved predominantly genes from the homologous recombination pathway (**Figure 6**) (59–61). Platinum-sensitive profile was characterized by higher methylation of *RAD50* and *RBBP8* genes, higher expression of *HELQ* and by identified somatic mutations in *BRCA1* gene (splice site and frame shift deletion variants) from HR pathway. This profile also included higher expression of *DDB2* and *MAD2L2* from NER pathway, respectively from DNA polymerase family and somatic mutations in *RAD9A* (missense variants), which is part of group of DNA damage response genes. Platinum-resistant profile was characterized by identified somatic mutations in *PRKDC* (NHEJ pathway), *XPC* (NER pathway) genes and higher expression of *PRPF19* gene, which is also part of at least in two DNA repair pathways.

## Discussion and conclusion

Here we investigated the complexity of regulatory aspects of gene expression of DNA repair machinery in EOC patients with different responses to adjuvant chemotherapy based on paclitaxel and platinum derivatives. Analysis of 178 genes covering the entire DNA repair machinery was conducted on three different levels: gene expression profile, methylation profile, and somatic genetic variability. The importance of alterations in DNA repair genes/pathways in the development and prognosis of ovarian cancer has recently been reviewed by us (60). The fact that mutations in *BRCA1/2* genes are associated with a higher risk of ovarian cancer has been utilized to give rise to one of the first molecular biomarkers in personalized medicine approved for clinical use (62). Patients with recurrent disease and carrying *BRCA1/2* mutations benefit from treatment based on PARPi inhibitors.

The evaluation of the association of expression profile of DNA repair genes with response to chemotherapy, based on PFI, revealed four differentially expressed genes between EOC patients with different platinum resistance status. In platinum-sensitive patients we found three upregulated (*DDB2, HELQ*, and *MAD2L2*) and one downregulated gene (*PRPF19*). *DDB2* (DNA damage-binding protein 2) gene is involved in the NER pathway, *HELQ* (Helicase, POLQ-like) in HR, *MAD2L2* (Mitotic Arrest Deficient 2 like 2) gene encodes DNA polymerase, and *PRPF19* (Pre-mRNA Processing Factor 19) gene is an integral component of DNA damage response, especially HR and NHEJ pathways (59, 61, 63). Nevertheless, those results from differential expression analysis did not pass the FDR test for multiple testing correction. Significant results passing FDR correction were found for the *DUT* expression. Higher levels of *DUT* gene were associated with the presence of EOC metastases, even that only 4 patients had them. *DUT* encodes Deoxyuridine 5’-triphosphate nucleotidohydrolase - an essential enzyme of nucleotide metabolism, involved in the metabolism of 5-fluorouracil (5-FU) (64, 65). Among other genes, DNA microarray analysis of 5-FU responsive genes identified *DUT* as a biomarker associated with resistance to 5-FU and also cisplatin in colorectal cancer patients (66). *DUT* RNA expression was also associated with shorter disease-free survival in colorectal cancer patients undergoing adjuvant chemotherapy with 5-FU (67). In gastric tumor patients, positive expression of DUT at the protein level was associated with high grade and younger age at diagnosis (68). Our study revealed for the first time the association of *DUT* expression with the presence of distant metastases, which are a sign of worse prognosis in EOC patients. These findings suggest that high *DUT* expression may be associated with worse prognosis and it may play role in the response to DNA damaging agents such as platinum derivatives used in EOC therapy.

The second part of the study explored the methylation profile of DNA repair genes in EOC therapy response. The differential methylation profile of the *RAD50* gene encoding the RAD50 double strand break repair protein, a member of the HR pathway, revealed higher methylation of the *RAD50* TSS1500 region in platinum-sensitive group of patients compared to platinum-resistant ones. DNA damage (intra-strand crosslinks) induced by platinum derivatives are repaired by NER pathway, while inter-strand crosslinks are repaired by interplay of NER and HR pathway (or other pathway repairing dsDNA breaks) (69). RAD50 is as member of MRN complex (MRE11-RAD50-NBS1) key component of HR pathway and plays an important role in the dsDNA break repair through HR pathway (70). Disrupted function of MRN complex leads to gene instability and accumulation of DNA damage, which can be demonstrated by observed association of MRE11 mutations with predisposition to colorectal cancer (71). Lower expression of MRE11 correlated with higher sensitivity to oxaliplatin treatment in colorectal cancer, together with significant reduction of tumor mass and longer progression-free survival (72). In the case of ovarian cancer, studies showing mutation rate of RAD50 is 0.12% (73). Immunohistochemical analysis of MRN complex showed that 41% of low-grade ovarian cancer tumors lacked MRN complex and that in 10.3% RAD50 tumors lacked its expression (74). Germline mutations of *RAD50* are known to be associated with a higher risk of breast carcinoma and its poor prognosis, whereas its role in ovarian cancer is still under investigation (75–77). Until now, studies focusing on the DNA methylation profile of *RAD50* in EOC are scarce in contrast to the *RAD50* gene expression. Kalra and Bapat found an increase of *RAD50* expression caused by cisplatin treatment in ovarian cancer cells (78). Recently, a higher basal mRNA expression and protein level of *RAD50* were found in platinum-resistant ovarian cancer A2780 and PEO4 cell lines and siRNA depletion of *RAD50* increased cisplatin sensitivity (79). A higher protein level of the MRE11-RAD50-NBS1 complex was also found in omental metastases of the EOC (80). These results suggest that higher expression of *RAD50* may be associated with the progression of EOC and its resistance to cisplatin. We recently summarized the *RAD50* roles in ovarian carcinogenesis, prognosis, and therapy response (60). Unfortunately, knowledge about *RAD50* methylation in ovarian cancer is limited, studies were dominantly focused on genetic variability and gene expression. So, *RAD50* gene expression, genetic variability, as well as methylation profile as was shown in our results, should be estimated in EOC patients as a factor affecting the therapeutic response. Another significant result based on the methylation profile was a positive correlation of the higher methylation profile of *POLL* with higher age. DNA polymerase λ, coded by *POLL*, is involved in BER and NHEJ pathways (81), required for cell cycle progression, and involved in the DNA damage checkpoint in the S phase of the cell cycle (82). Moreover, a possible role of *POLL* in telomere maintenance by the ALT mechanism was observed in osteosarcomas cell lines (Saos-2, U2OS) (83). Telomere length is frequently studied in aging and carcinogenesis (84).

Regarding methylation profile, the other interesting result was found for *RBBP8* (RB Binding Protein 8, endonuclease) in connection to chemotherapy response and the presence of somatic mutations in *BRCA1* and *RAD9A*. In particular, we observed a significantly higher level of *RBBP8* methylation in platinum-sensitive EOC patients harboring mutations in *BRCA1* (p.L1476fs and g.chr17:43076608delA) and *RAD9A* (p.E184K and p.G24R). *RBBP8* gene serves as an interactor between the *Rb* and *BRCA1* genes and acts in dsDNA breaks resection during the HR repair pathway (85). Two studies on bladder cancer samples showed a connection of *RBBP8* hypermethylation with longer overall survival (86) and with an increased HR deficient signature (87). The association of germline and somatic mutations in *RBBP8*, including LOH, with a worse prognosis in ovarian cancer patients, was also reported (88). However, knowledge about *RBBP8* gene methylation profile and its implication in ovarian cancer is limited. We found only two studies focusing on *RBBP8* methylation profile and these studies supporting our results on potential association of *RBBP8* higher methylation profile with better outcome for cancer patients, probably by biological interaction of *RBBP8* with *BRCA1* in HR pathway, which play role in platinum sensitivity. In summary, the methylation part of the present study disclosed two potential candidates for further testing in a larger cohort of EOC patients – *RAD50* (despite the result did not pass the FDR test) and *RBBP8* (in connection with simultaneous *BRCA1* or *RAD9A* somatic mutations).

Finally, integration analysis of somatic genetic variability with expression profiles showed interesting results for the presence of *XPC* (Xeroderma pigmentosum, complementation group C) and *PRKDC* (Protein Kinase, DNA-Activated, Catalytic Subunit) somatic mutations. Platinum-resistant EOC patients bearing *XPC* (rs750450365 and p.E433K) or *PRKDC* (p.E3448G, p.Y1243R, and p.L1242fs) mutations had significantly higher levels of *FAAP20* mRNA (FA Core Complex Associated Protein 20). This finding was confirmed by the TCGA dataset analysis. Additionally, survival analysis showed that the above-mentioned somatic mutations in *XPC* and *PRKDC* were associated with shorter overall survival in the present study. Association of *PRKDC* mutation with the survival of EOC patients has not been observed till now. To our best knowledge, no studies presenting results of genetic variability in *PRKDC* exist. On the other hand, data are available for highly polymorphic *XPC*. For example, the presence of rs2228001 in *XPC* was associated with a higher risk of EOC development (89), and the presence of rs3731108 and rs1124303 was associated with prolonged progression-free survival (90). To date, those variants are not included in the ClinVar database, which report the clinical significance of genetic variants. The expression of *XPC* and *PRKDC* genes was found to be associated with poor prognosis and worse survival in ovarian carcinoma patients (30, 91, 92). *In vitro* function studies revealed that knockdown of *PRKDC* enhanced the sensitivity of MCF7 breast cancer cell line to cisplatin (93) as well as cisplatin-resistant ovarian cancer cell lines SKOV3, PEO4, PEA2, PEO23, or A2780 (94, 95). Those studies support our data suggesting the importance of *XPC* and *PRKDC* deregulation and genetic variability for therapy response in EOC patients.

A modest sample size of our EOC set poses a limitation of this study. Due to this fact, rare (MAF = 1–5%) and very rare (MAF < 1%) variants detectable using the whole exome sequencing could have been missed. Larger validation studies need to be performed to confirm present observations resulting from WES, transcriptome, and methylation profiles of EOC patients. A limited number of patients precludes also any interactive studies, such as epistasis, correlations between gene variants, methylations and transcriptome. In addition, tumor heterogeneity and variabilities in treatment schemas would require larger sets of patients or group of patients selected for particular purpose. However, these studies are not feasible without the pilot investigation providing information for hypothesis building. Our ongoing research is now focused on the extension of our EOC set by addition of more patients or compiling with similarly designed set of patients with WES or RNA-seq and methylation array data. On the other hand, ethnical homogeneity and completeness of clinical follow up with defined PFI and sensitivity to EOC therapy can be considered the benefits of this study. Moreover, a large methylation profile using the new Infinium MethylationEPIC version of methylation array, covering over 850,000 methylation sites across the genome was determined and it is one the biggest strength of the present study. Together with well clinically characterized set of EOC patients with performing three robust techniques as WES and RNA sequencing with methylation profiling in the same set of patients. This comprehensive analysis of DNA repair gene methylation status allows us to reveal new and as of yet unknown intersections between gene methylation and transcriptome and genetic profile of DNA repair system genes in EOC patients. Functional studies of the identified variants and genes using CRISPR-Cas9 gene editing and subsequent gene function studies including response of the model cell line to clinically relevant drugs, e.g., taxanes will be the next step.

In conclusion, this study revealed for the first time several significant associations of DNA repair genes with prognosis and therapeutic response of EOC patients resulting from the integration of expression, methylation, and somatic genetic variability profiles. Namely, significant associations of *DUT* gene expression with the presence of EOC metastases represent unique observations. A survey of genetic variability in DNA repair genes confirmed highly mutated *TP53* in HGSC subtype of EOC patients. Using an exceptionally broad screen of the methylation profile, we found higher methylation of *RAD50* in platinum-sensitive EOC patients. Integration analyses revealed associations of somatic mutations in *BRCA1* and *RAD9A* with *RBBP8* methylation in sensitive compared to platinum-resistant EOC patients. In addition, we identified for the first time somatic mutations in *PRKDC* to be associated with sensitivity to therapy and overall survival of EOC patients. The presence of mutations *XPC*, a crucial gene involved in the NER pathway, is also associated with worse overall survival and may play an important role in the sensitivity to platinum-based ovarian cancer therapy. Results of our study need validation in larger cohorts of EOC patients with well-defined responses to adjuvant chemotherapy, homogeneous therapeutic regimens, and long-term follow-up.

## Data availability statement

The datasets presented in this study can be found in online repositories. The names of the repository/repositories and accession number(s) can be found below: https://www.ncbi.nlm.nih.gov/, BioProject ID: PRJNA814851 https://www.ncbi.nlm.nih.gov/, BioProject ID: PRJNA866991. Methylation data are available upon request to the corresponding author.

## Ethics statement

The studies involving human participants were reviewed and approved by Institutional Review Boards of the National Institute of Public Health in Prague, University Hospital Kralovske Vinohrady and University Hospital in Pilsen. The patients/participants provided their written informed consent to participate in this study.

## Author contributions

Conceptualization: RV and PV; investigation: KS, PH, VH, SB, TF, and MO; formal analysis, KS, TF, and PH; resources: LR, MH, MM, JB, and OF; visualization: KS; writing – original draft preparation: KS and RV; writing – review and editing: RV, PS, and PV; supervision: PS, LV, and VK. All authors contributed to the article and approved the submitted version.

## Funding

This study was supported by the Czech Science Foundation, project no. 19-10543S; the Ministry of Education, Youth and Sports, INTER-ACTION project no. LTAUSA19032, the Grant Agency of Charles University, project no. GAUK 1074120, the Czech Health Research Council-project no. NU20-09-00174, European Union’s Horizon 2020 research and innovation program, grant no. 856620 and Cooperatio program no. 207035, “Maternal and Childhood Care”, 3rd Faculty Medicine, Charles University.

## Acknowledgments

We would like to thank the Laboratory of Genomics and Bioinformatics (Institute of Molecular genetics of the Czech Academy of Sciences) and the Institute of Applied Biotechnologies for performing the sequencing analysis using the Illumina instruments (NextSeq 500 and NovaSeq 6000). The methylation array was performed at the Genomics Core Facility, Oslo University Hospital (http://oslo.genomics.no/).

## Conflict of interest

The authors declare that the research was conducted in the absence of any commercial or financial relationships that could be construed as a potential conflict of interest.

## Publisher’s note

All claims expressed in this article are solely those of the authors and do not necessarily represent those of their affiliated organizations, or those of the publisher, the editors and the reviewers. Any product that may be evaluated in this article, or claim that may be made by its manufacturer, is not guaranteed or endorsed by the publisher.
