# Supplementary material for: Complex molecular profile of DNA repair genes in epithelial ovarian carcinoma patients with different sensitivity to platinum-based therapy
Source: Front Oncol. 2022 Dec 2;12:1016958. doi: 10.3389/fonc.2022.1016958 (PMC9755737; doi:10.3389/fonc.2022.1016958)
Supplement: Supplementary file 1 [file DataSheet_1.pdf]

Supplementary Material

to

Complex molecular profile of DNA repair genes in epithelial ovarian carcinoma patient's with different sensitivity to platinum-based therapy

1 Supplementary Data

Table S1. Detailed clinical characteristics of OC patients from the TCGA database selected for the present study.

Table S2. Selection of 178 DNA repair genes for study.

Table S3. Probe coverage in the EPIC array for DNA repair genes followed in our study.

Table S4. Differential methylation analysis results. Results from differential methylation analysis at cg probe level.

Table S5. Differentially methylated genes and gene regions (TSS200, TSS1500, or promoter).

Table S6. Results of significant differences in DNA repair gene expression stratified by the presence of somatic mutations.

Table S7. Overview of significant potentially impactful somatic mutations in DNA repair genes.

Table S8. Associations of somatic mutations in DNA repair genes with *RBBP8* methylation.

Table S9. Top correlated DNA repair gene expression ($P < 0.01$) with methylation profile.

Figure S1. Differentially expressed gene *DUT* (deoxyuridine triphosphatase) stratified by the presence of peritoneal metastases (pM).

Figure S2. Differential *FAAP20* expression analysis stratified by the presence of somatic mutations in *PRKDC* and platinum-resistance status in the TCGA dataset.

Figure S3. Kaplan-Meier plots of overall survival for (**A**) *XPC* and (**B**) *PRKDC* somatic mutations.

Figure S4. Kaplan-Meier plots of overall survival for (**A**) *BRCA1-RAD9A-RAD50* and (**B**) *XPC-PRKDC-FAAP20* combinations.

2 Supplementary Tables

Patient´s characteristics from the TCGA database

We have selected patients from TCGA database based on characteristics of our dataset. The main criteria were adjuvant chemotherapy regimens corresponding to our patients set and information about response to the therapy. We used expression data from RNA sequencing expression profiling of 168 patients (N=168) and methylation microarrays for 232 patients (N=232).

The mean of patients´ age at diagnosis was 59.5 years for both cohorts. All patients´ tumor samples were histologically classified as Serous Cystadenocarcinoma (100 %) at stage III (77.9 %) and grade 3 (86.2 %) for all patients. Chemotherapy regimens combined mostly paclitaxel with carboplatin (88.3 %) or paclitaxel with cisplatin (6.3 %). The rest of the regimens combined paclitaxel with carboplatin and cisplatin (1.1 %) or involved platinum monotherapy (4.5 %). Platinum-free interval (PFI) was calculated from day of last dose of chemotherapy to the day of the relapse/death or the last follow up in remission. Based on PFI interval we classified patients as platinum-resistant (18.8 %) and platinum-sensitive ones (81.2 %). The mean PFI for platinum-sensitive patients was ~ 1313 - 1330 days and for platinum-resistant patients ~ 161.5 days.

Table S1. Detailed clinical characteristics of OC patients from the TCGA database selected for the present study.

**Characteristics**	**Expression profile** N (%)	**Methylation profile** N (%)
**Number of patients**	**168**	**232**
**Age** (mean ± SD, years)	59.8 ± 11.25	59.8 ± 11.58
**Stage**		
I	0 (0)	3 (1.3)
II	9 (5.4)	14 (6)
III	132 (78.6)	179 (77.2)
IV	27 (16)	36 (15.5)
**OC type**		
Serous Cystadenoma	168 (100)	232 (100)
**Histological Grade**		
G1	0 (0)	1 (0.4)
G2	18 (10.7)	23 (9.9)
G3	145 (86.3)	201 (86.6)
G4	1 (0.6)	5 (2.7)
GX	3 (1.8)	1 (0.4)
Not available	1 (0.6)	1 (0.6)

**Residuum after surgery**		
No Macroscopic Disease	29 (17.3)	49 (21.1)
> 20mm	40 (23.8)	51 (21.9)
11-20mm	13 (7.7)	16 (6.9)
1-10mm	80 (44.7)	99 (42.7)
Not available	11 (6.5)	17 (7.4)
**Regimen of chemotherapy**		
Paclitaxel with carboplatin	148 (88.1)	205 (88.4)
Paclitaxel with carboplatin and cisplatin	2 (1.2)	2 (0.9)
Paclitaxel with cisplatin	11 (6.5)	14 (6)
Platinum monotherapy	7 (4.2)	11 (4.7)
**Platinum resistance status**		
Platinum-sensitive	137 (81.5)	188 (81)
Platinum-resistant	31 (18.5)	44 (19)
**Treatment outcome first course - TCGA definition**		
Partial Remission/Response	20 (12)	25 (10.8)
Progressive Disease	14 (8.3)	22 (9.5)
Complete Remission/Response	95 (56.5)	126 (54.3)
Stable Disease	6 (3.6)	7 (3)
Not available	33 (19.6)	52 (22.4)
**Platinum-free interval (PFI)**		
All patients (mean ± SD; days)	1114 ± 851.5	1094.5 ± 845.4
Sensitive patients (mean ± SD; days)	1329.5 ± 796.4	1313 ± 803.9
Resistant patients (mean ± SD; days)	162 ± 122.8	160.8 ± 119

Footnotes:
SD (standard deviation)

Table S2. Selection of 178 DNA repair genes for study. DNA repair genes covering the whole DNA repair system. Selection of these genes was based on [58–60].

Gene Official Symbol	Alternative Names	Official Full Name
ALKBH2	*ABH2*	alkB homolog 2, alpha-ketoglutarate dependent dioxygenase
ALKBH3	*ABH3, PCA1, DEPC1, hABH3, DEPC-1*	alkB homolog 3, alpha-ketoglutarate dependent dioxygenase
APEX1	*APE, APX, APE1, APEN, APEX, HAP1, REF1*	apurinic/apyrimidinic endodeoxyribonuclease 1
APEX2	*APE2, XTH2, ZGRF2, APEXL2*	apurinic/apyrimidinic endodeoxyribonuclease 2
APLF	*APFL, PALF, Xip1, ZCCHH1, C2orf13*	aprataxin and PNKP like factor
APTX	*AOA, AOA1, AXA1, EAOH, EOAHA, FHA-HIT*	aprataxin
ATM	*AT1, ATA, ATC, ATD, ATE, ATDC, TEL1, TELO1*	ATM serine/threonine kinase
ATR	*FRP1, MEC1, SCKL, FCTCS, SCKL1*	ATR serine/threonine kinase
ATRIP		ATR interacting protein
BLM	*BS, RECQ2, RECQL2, RECQL3, MGRISCE1*	BLM RecQ like helicase
BRCA1	*IRIS, PSCP, BRCAI, BRCC1, FANCS, PNCA4, RNF53, BROVCA1, PPP1R53*	BRCA1 DNA repair associated
BRCA2	*FAD, FACD, FAD1, GLM3, BRCC2, FANCD, PNCA2, FANCD1, XRCC11, BROVCA2*	BRCA2 DNA repair associated
BRIP1	*OF, BACH1, FANCJ*	BRCA1 interacting helicase 1
SLX4	*FANCP, BTBD12, MUS312*	SLX4 structure-specific endonuclease subunit
CCNH	*CAK, p34, p37, CycH*	cyclin H
CDK7	*CAK, CAK1, HCAK, MO15, STK1, CDKN7, p39MO15*	cyclin dependent kinase 7
CETN2	*CALT, CEN2*	centrin 2
CLK2		CDC like kinase 2
DCLRE1A	*PSO2, SNM1, SNM1A*	DNA cross-link repair 1A
DCLRE1B	*SNM1B, SNMIB, APOLLO*	DNA cross-link repair 1B
DCLRE1C	*SCIDA, SNM1C, A-SCID, RS-SCID, DCLREC1C*	DNA cross-link repair 1C
DDB1	*XPE, DDBA, XAP1, XPCE, XPE-BF, UV-DDB1*	damage specific DNA binding protein 1
DDB2	*XPE, DDBB, UV-DDB2*	damage specific DNA binding protein 2
DMC1	*DMC1H, LIM15, dJ199H16.1*	DNA meiotic recombinase 1

DUT	*dUTPase*	deoxyuridine triphosphatase
EME1	*MMS4L, SLX2A*	essential meiotic structure-specific endonuclease 1
EME2	*SLX2B, gs125*	essential meiotic structure-specific endonuclease subunit 2
ENDOV		endonuclease V
ERCC1	*UV20, COFS4, RAD10*	ERCC excision repair 1, endonuclease non-catalytic subunit
ERCC2	*EM9, TTD, XPD, TTD1, COFS2, TFIIH*	ERCC excision repair 2, TFIIH core complex helicase subunit
ERCC3	*XPB, BTF2, Ssl2, TTD2, GTF2H, RAD25, TFIIH*	ERCC excision repair 3, TFIIH core complex helicase subunit
ERCC4	*XPF, RAD1, FANCQ, XFEPS, ERCC11*	ERCC excision repair 4, endonuclease catalytic subunit
ERCC5	*XPG, UVDR, XPGC, COFS3, ERCM2, ERCC5-201*	ERCC excision repair 5, endonuclease
ERCC6	*CSB, CKN2, COFS, ARMD5, COFS1, POF11, RAD26, UVSS1, CSB-PGBD3*	ERCC excision repair 6, chromatin remodeling factor
ERCC8	*CSA, CKN1, UVSS2*	ERCC excision repair 8, CSA ubiquitin ligase complex subunit
EXO1	*HEX1, hExoI*	exonuclease 1
FAAP20	*FP7162, C1orf86*	FA core complex associated protein 20
FAAP24	*C19orf40*	FA core complex associated protein 24
FAN1	*KMIN, hFAN1, MTMR15, KIAA1018*	FANCD2 and FANCI associated nuclease 1
FANCA	*FA, FA1, FAA, FAH, FA-H, FACA, FANCH*	FA complementation group A
FANCB	*FA2, FAB, FACB, FAAP90, FAAP95*	FA complementation group B
FANCC	*FA3, FAC, FACC*	FA complementation group C
FANCD2	*FA4, FAD, FACD, FAD2, FA-D2, FANCD*	FA complementation group D2
FANCE	*FAE, FACE*	FA complementation group E
FANCF	*FAF*	FA complementation group F
FANCG	*FAG, XRCC9*	FA complementation group G
FANCI	*KIAA1794*	FA complementation group I
FANCL	*POG, PHF9, FAAP43*	FA complementation group L
FANCM	*POF15, SPGF28, FAAP250, KIAA1596*	FA complementation group M
FEN1	*MF1, RAD2, FEN-1*	flap structure-specific endonuclease 1
GEN1	*Gen*	GEN1 Holliday junction 5' flap endonuclease

SLX1A	*GIYD1*	SLX1 homolog A, structure-specific endonuclease subunit
SLX1B	*GIYD2*	SLX1 homolog B, structure-specific endonuclease subunit
GTF2H1	*P62, BTF2, TFB1, TFIIH*	general transcription factor IIH subunit 1
GTF2H2	*p44, BTF2, TFIIH, BTF2P44, T-BTF2P44*	general transcription factor IIH subunit 2
GTF2H3	*P34, BTF2, TFB4, TFIIH*	general transcription factor IIH subunit 3
GTF2H4	*P52, TFB2, TFIIH*	general transcription factor IIH subunit 4
GTF2H5	*TTD, TFB5, TTD3, TTDA, TFIIH, TTD-A, TGF2H5, C6orf175, bA120J8.2*	general transcription factor IIH subunit 5
H2AX	*H2A.X, H2A/X, H2AFX*	H2A.X variant histone
HELQ	*HEL308*	helicase, POLQ like
HLTF	*ZBU1, HLTF1, RNF80, HIP116, SNF2L3, HIP116A, SMARCA3*	helicase like transcription factor
HUS1	*hHUS1*	HUS1 checkpoint clamp component
CHAF1A	*CAF1, P150, CAF-1, CAF1B, CAF1P150*	chromatin assembly factor 1 subunit A
CHEK1	*CHK1*	checkpoint kinase 1
CHEK2	*CHK2*	checkpoint kinase 2
LIG1		DNA ligase 1
LIG3	*LIG2, LIG3alpha*	DNA ligase 3
LIG4	*LIG4S*	DNA ligase 4
MAD2L2	*REV7, FANCV, MAD2B, POLZ2*	mitotic arrest deficient 2 like 2
MBD4	*MED1*	methyl-CpG binding domain 4, DNA glycosylase
MDC1	*NFBD1*	mediator of DNA damage checkpoint 1
MGMT		O-6-methylguanine-DNA methyltransferase
MLH1	*FCC2, COCA2, HNPCC, hMLH1, HNPCC2, MMRCS1*	mutL homolog 1
MLH3	*HNPCC7*	mutL homolog 3
MMS19	*CIAO4, MET18, MMS19L, hMMS19*	MMS19 homolog, cytosolic iron-sulfur assembly component
MNAT1	*MAT1, TFB3, CAP35, RNF66*	MNAT1 component of CDK activating kinase

MPG	*AAG, MDG, ADPG, APNG, Mid1, anpg, PIG11, PIG16, CRA36.1*	N-methylpurine DNA glycosylase
MRE11	*ATLD, HNGS1, MRE11A, MRE11B*	MRE11 homolog, double strand break repair nuclease
MSH2	*FCC1, COCA1, HNPCC, LCFS2, hMSH2, HNPCC1, MMRCS2*	mutS homolog 2
MSH3	*DUP, FAP4, MRP1*	mutS homolog 3
MSH4		mutS homolog 4
MSH5	*G7, MUTSH5, NG23, POF13*	mutS homolog 5
MSH6	*GTBP, GTMBP, HNPCC5, HSAP, MMRCS3, p160*	mutS homolog 6
MUS81	*SLX3*	MUS81 structure-specific endonuclease subunit
MUTYH	*MYH*	mutY DNA glycosylase
NBN	*ATV, NBS, P95, NBS1, AT-V1, AT-V2*	nibrin
NEIL1	*FPG1, NEI1, hFPG1*	nei like DNA glycosylase 1
NEIL2	*NEH2, NEI2*	nei like DNA glycosylase 2
NEIL3	*FGP2, FPG2, NEI3, ZGRF3, hFPG2, hNEI3*	nei like DNA glycosylase 3
NHEJ1	*XLF*	non-homologous end joining factor 1p
NTHL1	*FAP3, NTH1, OCTS3, hNTH1*	nth like DNA glycosylase 1
NUDT1	*MTH1*	nudix hydrolase 1
NABP2	*SSB1, hSSB1, OBFC2B, SOSS-B1*	nucleic acid binding protein 2
OGG1	*HMMH, MUTM, OGH1, HOGG1*	8-oxoguanine DNA glycosylase
PALB2	*FANCN, PNCA3*	partner and localizer of BRCA2
PARP1	*PARP, PPOL, ADPRT, ARTD1, ADPRT1, PARP-1, ADPRT 1, pADPRT-1*	poly(ADP-ribose) polymerase 1
PARP2	*ARTD2, ADPRT2, PARP-2, ADPRTL2, ADPRTL3, pADPRT-2*	poly(ADP-ribose) polymerase 2
PARP3	*IRT1, ARTD3, ADPRT3, ADPRTL2, ADPRTL3, PADPRT-3*	poly(ADP-ribose) polymerase family member 3
PCNA	*ATLD2*	proliferating cell nuclear antigen
PER1	*PER, hPER, RIGUI*	period circadian regulator 1
PMS1	*MLH2, PMSL1, hPMS1, HNPCC3*	PMS1 homolog 1, mismatch repair system component
PMS2	*MLH4, PMSL2, HNPCC4, MMRCS4, PMS2CL*	PMS1 homolog 2, mismatch repair system componen
PMS2P3	*PMS5, PMSR3, PMS2L3, PMS2L9*	PMS1 homolog 2, mismatch repair system component pseudogene 3
PNKP	*PNK, AOA4, MCSZ, CMT2B2, EIEE10*	polynucleotide kinase 3'-phosphatase
POLB		DNA polymerase beta

POLD1	*CDC2, MDPL, POLD, CRCS10*	DNA polymerase delta 1, catalytic subunit
POLE	*FILS, POLE1, CRCS12, IMAGEI*	DNA polymerase epsilon, catalytic subunit
POLG	*PEO, MDP1, SCAE, MIRAS, POLG1, POLGA, SANDO, MTDPS4A, MTDPS4B*	DNA polymerase gamma, catalytic subunit
POLH	*XPV, XP-V, RAD30, RAD30A*	DNA polymerase eta
POLI	*eta2, RAD30B, RAD3OB*	DNA polymerase iota
POLK	*DINP, POLQ, DINB1*	DNA polymerase kappa
POLL	*BETAN, POLKAPPA*	DNA polymerase lambda
POLM	*Tdt-N, Pol Mu*	DNA polymerase mu
POLN	*POL4P*	DNA polymerase nu
POLQ	*PRO0327*	DNA polymerase theta
PRKDC	*HYRC, p350, DNAPK, DNPK1, HYRC1, IMD26, XRCC7, DNAPKc, DNA-PKC, DNA-PKcs*	protein kinase, DNA-activated, catalytic subunit
PRPF19	*PSO4, SNEV, PRP19, UBOX4, hPSO4, NMP200*	pre-mRNA processing factor 19
RAD1	*REC1, HRAD1*	RAD1 checkpoint DNA exonuclease
RAD17	*CCYC, R24L, RAD24, HRAD17, RAD17SP*	RAD17 checkpoint clamp loader component
RAD18	*RNF73*	RAD18 E3 ubiquitin protein ligase
RAD23A	*HR23A, HHR23A*	RAD23 homolog A, nucleotide excision repair protein
RAD23B	*P58, HR23B, HHR23B*	RAD23 homolog B, nucleotide excision repair protein
RAD50	*NBSLD, RAD502, hRad50*	RAD50 double strand break repair protein
RAD51	*RECA, BRCC5, FANCR, MRMV2, HRAD51, RAD51A, HsRad51, HsT16930*	RAD51 recombinase
RAD51B	*REC2, R51H2, RAD51L1*	RAD51 paralog B
RAD51C	*FANCO, R51H3, BROVCA3, RAD51L2*	RAD51 paralog C
RAD51D	*TRAD, R51H3, BROVCA4, RAD51L3*	RAD51 paralog D
RAD52		RAD52 homolog, DNA repair protein
RAD54B	*RDH54*	RAD54 homolog B
RAD54L	*HR54, hHR54, RAD54A, hRAD54*	RAD54 like
RAD9A	*RAD9*	RAD9 checkpoint clamp component A
RBBP8	*RIM, COM1, CTIP, JWDS, SAE2, SCKL2*	RB binding protein 8, endonuclease
RDM1	*RAD52B*	RAD52 motif containing 1

RECQL	*RecQ1, RECQL1*	RecQ like helicase
RECQL4	*RECQ4*	RecQ like helicase 4
RECQL5	*RECQ5*	RecQ like helicase 5
REV1	*REV1L, AIBP80*	REV1 DNA directed polymerase
REV3L	*POLZ, REV3*	REV3 like, DNA directed polymerase zeta catalytic subunit
RIF1		replication timing regulatory factor 1
RNF168	*RIDL, hRNF168*	ring finger protein 168
RNF4	*SLX5, SNURF, RES4-26*	ring finger protein 4
RNF8	*hRNF8*	ring finger protein 8
RPA1	*HSSB, RF-A, RP-A, REPA1, RPA70, MST075*	replication protein A1
RPA2	*REPA2, RPA32, RP-A p32, RP-A p34*	replication protein A2
RPA3	*REPA3, RP-A p14*	replication protein A3
RPA4	*HSU24186*	replication protein A4
RRM2B	*P53R2, MTDPS8A, MTDPS8B*	ribonucleotide reductase regulatory TP53 inducible subunit M2B
SETMAR	*Mar1, METNASE*	SET domain and mariner transposase fusion gene
SEM1	*ECD, DSS1, SHFD1, SHFM1, SHSF1, PSMD15, Shfdg1, C7orf76*	SEM1 26S proteasome subunit
SHPRH	*bA545I5.2*	SNF2 histone linker PHD RING helicase
SMUG1	*FDG, UNG3, HMUDG*	single-strand-selective monofunctional uracil-DNA glycosylase 1
SPO11	*CT35, TOPVIA, SPATA43, TOPOVIA*	SPO11 initiator of meiotic double stranded breaks
SPRTN	*DVC1, PRO4323, spartan, C1orf124*	SprT-like N-terminal domain
TDG	*hTDG*	thymine DNA glycosylase
TDP1		tyrosyl-DNA phosphodiesterase 1
TDP2	*EAP2, AD022, EAPII, TTRAP, hTDP2, dJ30M3.3*	tyrosyl-DNA phosphodiesterase 2
TOPBP1	*Dpb11, TOP2BP1*	DNA topoisomerase II binding protein 1
TP53	*P53, BCC7, LFS1, BMFS5, TRP53*	tumor protein p53
TP53BP1	*p202, 53BP1, TDRD30, p53BP1*	tumor protein p53 binding protein 1
TREX1	*CRV, AGS1, DRN3, HERNS, RVCLS*	three prime repair exonuclease 1
TREX2		three prime repair exonuclease 2
MPLKIP	*ABHS, TTD4, ORF20, C7orf11*	M-phase specific PLK1 interacting protein

UBE2A	*UBC2, HHR6A, MRXSN, RAD6A, MRXS30*	ubiquitin conjugating enzyme E2 A
UBE2B	*HR6B, UBC2, HHR6B, RAD6B, E2-17kDa*	ubiquitin conjugating enzyme E2 B
UBE2N	*UBC13, UbcH13, HEL-S-71, UbcH-ben, UBCHBEN, UBC13*	ubiquitin conjugating enzyme E2 N
UBE2V2	*MMS2, UEV2, EDPF1, UEV-2, DDVIT1, EDAF-1, EDPF-1, DDVit-1*	ubiquitin conjugating enzyme E2 V2
UNG	*DGU, UDG, UNG1, UNG2, HIGM4, HIGM5, UNG15*	uracil DNA glycosylase
UVSSA	*UVSS3, KIAA1530*	UV stimulated scaffold protein A
WRN	*RECQ3, RECQL2, RECQL3*	WRN RecQ like helicase
XAB2	*HCNP, HCRN, SYF1, NTC90*	XPA binding protein 2
XPA	*XP1, XPAC*	XPA, DNA damage recognition and repair factor
XPC	*XP3, RAD4, XPCC, p125*	XPC complex subunit, DNA damage recognition and repair factor
XRCC1	*RCC, SCAR26*	X-ray repair cross complementing 1
XRCC2	*FANCU, POF17, SPGF50*	X-ray repair cross complementing 2
XRCC3	*CMM6*	X-ray repair cross complementing 3
XRCC4	*SSMED*	X-ray repair cross complementing 4
XRCC5	*KU80, KUB2, Ku86, NFIV, KARP1, KARP-1*	X-ray repair cross complementing 5
XRCC6	*ML8, KU70, TLAA, CTC75, CTCBF, G22P1*	X-ray repair cross complementing 6

Table S3. Probe coverage in the EPIC array for DNA repair genes followed in our study. Some probes overlap between gene regions based on their genomic localization.

Gene	Whole gene	TSS200	TSS1500	5´UTR	1stExon	Body	ExonBoundary	3´UTR
ALKBH2	20	4	4	7	4	9	0	0
ALKBH3	30	3	5	5	1	16	0	1
APEX1	16	2	5	7	5	3	0	0
APEX2	12	2	4	2	3	2	0	0
APLF	26	5	6	2	2	13	0	0
APTX	18	5	4	10	4	11	0	0
ATM	71	6	33	6	4	23	1	1
ATR	30	5	5	2	2	18	0	0
ATRIP	27	5	7	2	3	15	1	0
BLM	46	5	5	17	3	29	1	1
BRCA1	50	14	35	18	11	16	1	1
BRCA2	21	3	4	4	0	9	1	1
BRIP1	25	4	7	2	1	11	0	1
BTBD12	19	0	1	3	0	14	0	1
CCNH	20	5	5	5	4	10	1	0
CDK7	19	5	3	5	5	6	0	0
CETN2	19	4	9	0	0	5	0	1
CLK2	31	6	4	7	3	14	0	2
DCLRE1A	24	13	12	5	5	5	0	1
DCLRE1B	17	0	9	1	1	4	0	1
DCLRE1C	35	7	3	8	2	16	0	1
DDB1	39	2	8	6	6	23	2	1
DDB2	17	1	5	1	1	9	0	1
DMC1	16	0	9	8	3	3	0	0
DUT	27	8	15	14	6	15	0	0
EME1	23	3	6	9	3	3	1	2
EME2	20	3	5	0	3	9	1	0
ENDOV	16	0	0	0	0	16	2	1
ERCC1	20	10	7	7	3	2	0	2
ERCC2	25	2	2	4	4	18	0	1
ERCC3	16	4	2	1	2	7	0	1
ERCC4	31	4	5	0	1	19	0	2
ERCC5	31	6	3	1	2	20	1	0

ERCC6	52	6	9	5	1	31	0	1
ERCC8	24	4	3	5	4	12	0	1
EXO1	19	7	7	10	4	4	0	1
FAAP20	27	5	6	13	2	13	1	4
FAAP24	1	0	0	0	0	0	0	1
FAN1	12	1	1	1	1	10	1	0
FANCA	46	0	8	0	0	37	3	2
FANCB	17	5	6	5	2	1	0	0
FANCC	80	1	4	20	1	49	0	1
FANCD2	36	4	5	8	1	18	2	1
FANCE	12	3	4	1	1	3	0	1
FANCF	24	8	7	1	10	0	0	1
FANCG	19	6	4	2	3	5	0	1
FANCI	24	4	5	5	1	10	1	0
FANCL	22	4	3	2	3	11	1	1
FANCM	31	4	11	1	4	11	1	0
FEN1	26	7	5	10	6	3	0	1
GEN1	25	2	7	11	4	8	1	1
GTF2H1	32	5	5	9	4	12	0	1
GTF2H3	29	8	7	5	1	12	0	1
GTF2H4	56	1	4	4	3	41	0	6
GTF2H5	22	6	5	7	0	3	0	1
H2AX	12	5	3	2	4	0	0	1
HELQ	17	5	5	5	3	5	0	0
HLTF	36	5	17	2	2	11	1	1
HUS1	16	3	1	1	1	9	1	1
CHAF1A	22	1	4	0	1	16	0	0
CHEK1	27	4	9	12	8	15	1	4
CHEK2	26	4	6	7	1	8	1	0
LIG1	35	2	4	9	5	24	1	1
LIG3	19	5	6	2	1	4	0	2
LIG4	28	11	14	21	6	0	0	0
MAD2L2	37	6	10	27	7	3	1	1
MBD4	29	9	5	7	9	6	1	0
MDC1	64	4	10	33	17	16	2	1

MGMT	165	4	18	1	1	139	0	3
MLH1	55	15	30	14	6	22	0	1
MLH3	28	3	1	4	1	19	1	1
MMS19	22	5	9	2	1	13	2	0
MNAT1	23	5	3	1	1	30	1	0
MPG	21	7	13	9	2	6	0	0
MPLKIP	14	5	5	3	2	1	1	1
MRE11	14	3	4	7	4	10	0	1
MSH2	19	2	5	1	2	16	0	0
MSH3	16	1	3	2	2	29	0	0
MSH4	15	1	6	1	2	6	0	0
MSH5	19	8	12	11	8	31	1	1
MSH6	15	3	6	2	2	4	0	0
MUS81	19	4	9	1	1	14	3	3
MUTYH	24	12	15	21	8	18	3	0
NABP2	19	5	3	5	5	5	0	1
NBN	17	5	5	3	1	8	0	0
NEIL1	33	7	11	15	6	14	0	0
NEIL2	19	5	4	7	4	2	0	1
NEIL3	24	6	2	0	3	12	0	1
NHEJ1	33	4	5	7	0	17	0	2
NTHL1	30	2	6	0	2	17	0	3
NUDT1	30	6	12	7	2	11	0	1
OGG1	29	5	5	5	6	12	0	5
PALB2	28	5	5	3	4	14	1	0
PARP1	35	3	2	3	5	24	2	1
PARP2	18	2	2	0	3	13	3	0
PARP3	24	5	8	4	2	8	0	1
PCNA	35	12	10	20	5	3	0	0
PER1	18	4	5	2	0	6	0	1
PMS1	37	4	7	16	10	21	2	0
PMS2	15	0	2	3	3	13	0	0
PMS2L3	11	6	2	0	0	3	0	0
PNKP	14	1	2	2	1	8	0	1
POLB	18	3	5	1	1	9	0	0

POLD1	25	2	5	6	1	14	0	0
POLE	55	4	1	2	2	75	1	0
POLG	33	3	9	2	2	21	1	0
POLH	21	3	6	9	4	6	0	1
POLI	21	5	3	0	1	7	0	1
POLK	41	2	6	24	1	9	1	0
POLL	29	5	7	6	2	14	0	1
POLM	19	4	4	1	2	8	1	1
POLN	52	2	3	5	2	41	1	1
POLQ	37	5	6	3	4	21	0	1
PRKDC	78	4	7	0	2	64	2	1
PRPF19	17	5	5	1	1	5	0	1
RAD1	23	4	7	8	3	14	0	1
RAD17	40	9	27	16	2	6	1	4
RAD18	26	4	5	0	0	15	0	2
RAD23A	16	5	5	2	2	4	0	0
RAD23B	26	6	8	12	9	17	0	1
RAD50	29	4	5	5	3	17	2	0
RAD51	19	7	5	4	3	4	0	1
RAD51B	169	2	2	3	1	162	0	0
RAD51C	22	2	10	0	0	10	0	0
RAD51D	8	1	2	0	0	5	1	0
RAD52	34	9	2	13	1	19	2	0
RAD54B	29	6	6	5	2	18	0	0
RAD54L	21	2	4	6	6	9	1	0
RAD9A	23	3	7	1	2	12	1	2
RBBP8	19	5	8	6	2	6	0	0
RDM1	14	4	8	1	4	10	0	1
RECQL	17	2	6	6	5	4	1	0
RECQL4	18	4	6	0	0	10	0	0
RECQL5	62	3	5	5	5	49	0	3
REV1	28	0	3	3	1	22	1	0
REV3L	47	3	9	10	3	29	1	0
RIF1	19	6	2	3	1	9	1	1
RNF168	22	4	6	2	4	7	0	1

RNF4	35	7	6	15	1	9	1	1
RNF8	23	3	5	2	3	11	0	1
RPA1	46	0	4	0	0	35	1	8
RPA2	19	6	5	3	3	4	0	1
RPA3	29	0	1	21	1	5	0	1
RPA4	5	2	2	1	1	0	0	0
RRM2B	19	5	4	5	5	6	0	1
SEM1	13	4	4	1	2	3	0	0
SETMAR	18	8	4	1	4	7	0	0
SHPRH	30	5	8	6	2	12	1	1
SLX1A	7	0	0	0	0	7	1	0
SLX1B	7	0	0	0	0	7	1	0
SMUG1	28	6	4	11	1	12	1	1
SPO11	19	5	3	2	1	9	0	0
SPRTN	11	1	2	4	4	4	0	0
TDG	19	3	3	3	2	9	0	1
TDP1	30	1	9	12	2	8	0	0
TDP2	7	1	1	0	1	4	0	0
TOPBP1	25	6	5	4	1	10	1	0
TP53	42	4	15	22	5	7	1	1
TP53BP1	48	16	9	13	5	18	1	0
TREX1	18	3	7	6	7	1	0	1
TREX2	17	6	5	2	1	5	0	0
UBE2A	20	6	5	4	4	5	0	1
UBE2B	18	5	3	1	1	8	0	1
UBE2N	28	5	5	5	4	12	0	2
UBE2V2	25	4	6	0	0	14	1	1
UNG	28	9	8	4	5	12	1	3
UVSSA	25	0	0	2	2	24	0	0
WRN	37	3	9	21	8	4	0	1
XAB2	20	6	1	0	2	13	0	1
XPA	8	3	0	2	2	4	0	0
XPC	33	7	3	2	2	20	2	1
XRCC1	26	8	6	2	1	13	0	0
XRCC2	12	4	2	1	2	5	0	0

XRCC3	55	5	6	16	1	25	0	2
XRCC4	38	5	4	11	6	15	0	0
XRCC5	29	2	4	1	1	21	1	1
XRCC6	34	3	8	11	0	11	1	0

Table S4. Differential methylation analysis results. Results from differential methylation analysis at cg probe level. In total analysis, 27 cg probes with higher methylation profile in platinum-sensitive patients (positive logFC, red color) and 23 cg probes with lower methylation profile compared to platinum-resistant patients (negative logFC, blue color) shown.

Probe	Gene	logFC	P-value
cg17991919	*RAD50*	1.43	3.724×10^{-06}
cg06340416	*RAD50*	1.11	5.971×10^{-05}
cg05258927	*RAD50*	1.31	1.0×10^{-04}
cg05198819	*RAD50*	0.80	6.0×10^{-04}
cg08207536	*FANCD2*	0.48	7.0×10^{-04}
cg02201766	*RAD50*	0.61	9.0×10^{-04}
cg10461218	*GTF2H3*	-0.56	0.001
cg16078412	*NHEJ1*	0.64	0.001
cg08110332	*MBD4*	-0.26	0.002
cg02762573	*RAD51C*	-0.36	0.002
cg26467754	*RECQL4*	-0.45	0.002
cg02777063	*RPA3*	-0.25	0.002
cg01916724	*PARP3*	0.41	0.003
cg26975850	*POLK*	-0.64	0.003
cg02700841	*PRKDC*	-0.63	0.003
cg22996031	*RAD9A*	0.52	0.003
cg08136893	*C7orf11*	-0.21	0.003
cg07550334	*MLH3*	-0.16	0.004
cg18538644	*PRKDC*	-0.22	0.004
cg11714602	*RAD50*	-0.64	0.004
cg15079957	*RNF4*	-0.68	0.004
cg24422008	*PARP1*	-0.18	0.004
cg14816013	*OBFC2B*	-0.19	0.004
cg02447314	*POLI*	-0.29	0.005
cg20674128	*ERCC2*	0.68	0.005
cg20537325	*MGMT*	0.89	0.005
cg24054272	*CLK2*	-0.26	0.005
cg06084702	*MAD2L2*	0.61	0.005
cg07386086	*PRKDC*	-0.78	0.006
cg00711252	*CHEK2*	0.66	0.006
cg16134717	*MAD2L2*	0.89	0.006

cg11514965	*RAD51B*	-0.70	0.006
cg13284983	*PER1*	0.42	0.007
cg22492943	*LIG4*	-0.19	0.007
cg17726328	*ATM*	-0.56	0.007
cg02021919	*CCNH*	0.62	0.007
cg16003913	*MPG*	0.46	0.007
cg21226234	*RECQL4*	0.32	0.007
cg01702039	*POLE*	-0.58	0.008
cg19229566	*NEIL1*	0.22	0.008
cg26959303	*TOPBP1*	-0.33	0.008
cg14597804	*RAD50*	-0.59	0.008
cg13619810	*RPA3*	-0.20	0.008
cg14200364	*MSH4*	0.61	0.009
cg27056559	*MGMT*	0.85	0.009
cg18511205	*EME1*	0.41	0.009
cg15559074	*ALKBH3*	0.60	0.009
cg16158782	*MSH3*	0.55	0.009
cg09475882	*TOPBP1*	0.29	0.009
cg08279097	*DCLRE1B*	0.63	0.009

Table S5. Differentially methylated genes and gene regions (TSS200, TSS1500, or promoter). Analysis showed higher methylation profile of DNA repair genes regions for all these genes in patients with resistant status (positive logFC, red color). Only for *RAD50* (TSS1500 region) and *XRCC4* (whole gene) methylation profile was higher in EOC patients with platinum-sensitive status (negative logFC, blue color).

Gene	logFC	P-value
**TSS200**		
MLH3	0.16	0.001
MRE11A	0.17	0.007
GTF2H3	0.13	0.025
HLTF	0.27	0.026
CCNH	0.13	0.049
**TSS1500**		
PMS2L3	0.10	0.007
RAD50	-0.70	0.007
SHFM1	0.50	0.014
DMC1	0.41	0.024
FANCG	0.69	0.034
CHEK2	0.10	0.035
MRE11A	0.16	0.036
PARP3	0.13	0.037
RAD9A	0.14	0.042
TP53BP1	0.11	0.043
**Promoter**		
MLH3	0.16	0.001
MRE11A	0.17	0.007
GTF2H3	0.13	0.025
HLTF	0.27	0.025
CCNH	0.13	0.049
**Whole gene**		
XRCC4	-0.32	0.045

Table S6. Overview of significant potentially impactful somatic mutations in DNA repair genes.

Gene	Sample ID	Variant classification	Variant type	dbSNP (v151) accession no.	Genome change	Protein change	Chrom.	Start Position	End position
BRCA1	Sample no. 28	Splice Site	SNV	rs80358041	g.chr17:43051062C>T	p.L1476fs	chr17	43051062	43051062
	Sample no. 48	Frame Shift Del	DEL		g.chr17:43076608delA		chr17	43076608	43076608
PRKDC	Sample no. 25	Missense	SNV		g.chr8:47798352T>C	p.E3448G	chr8	47798352	47798352
	Sample no. 39	Missense	DNV		g.chr8:47893258_47893259TA >CG	p.Y1243R	chr8	47893258	47893259
	Sample no. 39	Frame Shift Ins	INS		g.chr8:47893263_47893264ins CCAGCTGCAAATGCAAAT GCCATTATATTTAAAATCA GACGACATAACAC	p.L1242fs	chr8	47893263	47893264
	TCGA-13-0795	Missense	SNV			p.K2716R	chr8	48746759	48746759
	TCGA-13-0899	Frame Shift Ins	INS			p.G3646A fs*4	chr8	48697841	48697842
	TCGA-13-1477	Missense	SNV			p.A2960T	chr8	48736460	48736460
	TCGA-61-2110	Missense	SNV			p.V3600L	chr8	48701568	48701568
	TCGA-09-2044	Missense	SNV			p.P3972Q	chr8	48690371	48690371
	TCGA-24-1565	Frame Shift Del	DEL			p.K2220N fs*18	chr8	48767881	48767881
RAD9A	Sample no. 28	Missense	SNV	rs754732107	g.chr11:67395816G>A	p.E184K	chr11	67395816	67395816
	Sample no. 5	Missense	SNV	rs1360441369	g.chr11:67392196G>A	p.G24R	chr11	67392196	67392196
TP53	Sample no. 25	Missense	SNV	rs1057519992	g.chr17:7674890T>C	p.H214R	chr17	7674890	7674890
	Sample no. 56/no. 14	Missense	SNV	rs28934576	g.chr17:7673802C>T	p.R273H	chr17	7673802	7673802

Sample no. 48	Missense	SNV	rs483352695	g.chr17:7674227T>C	p.M246V	chr17	7674227	7674227
Sample no. 28	Splice Site	SNV	rs80358041	g.chr17:43051062C>T	p.Y163C	chr17	43051062	43051062
Sample no. 39/no. 23/no. 27	Nonsense	SNV	rs397516436	g.chr17:7674894G>A	p.R213*	chr17	7674894	7674894
Sample no. 62	Missense	SNV	rs786202962	g.chr17:7675085C>A	p.C176F	chr17	7675085	7675085
Sample no. 68	Missense	SNV	rs28934574	g.chr17:7673776G>A	p.R282W	chr17	7673776	7673776
Sample no. 69/no. 71/no. 17/no.21/no. 10	Missense	SNV	rs28934578	g.chr17:7675088C>T	p.R175H	chr17	7675088	7675088
Sample no. 61	Frame Shift Ins	INS		g.chr17:7673582_7673583insA	p.P316fs	chr17	7673582	7673583
Sample no. 18	Missense	SNV	rs1057519976	g.chr17:7675207G>C	p.C135W	chr17	7675207	7675207
Sample no. 19	Missense	SNV	rs876660754	g.chr17:7675095C>A	p.V173L	chr17	7675095	7675095
Sample no. 59	Missense	SNV	rs1057519996	g.chr17:7675217T>C	p.K132R	chr17	7675217	7675217
Sample no. 59	Missense	SNV	rs1057519991	g.chr17:7675076T>C	p.H179R	chr17	7675076	7675076
Sample no. 2	Frame Shift Del	DEL		g.chr17:7670694_7670695delCG	p.F338fs	chr17	7670694	7670695
Sample no. 31/no.1	Missense	SNV	rs11540652	g.chr17:7674220C>T	p.R248Q	chr17	7674220	7674220
Sample no. 32	Missense	SNV		g.chr17:7673806C>G	p.V272L	chr17	7673806	7673806
Sample no. 37	Missense	SNV		g.chr17:7670702T>C	p.E336G	chr17	7670702	7670702
Sample no. 37	Missense	SNV		g.chr17:7674899G>A	p.T211I	chr17	7674899	7674899
Sample no. 38	Frame Shift Del	DEL	rs864309495	g.chr17:7674895delA	p.R213fs	chr17	7674895	7674895
Sample no. 41	Missense	SNV	rs786201059	g.chr17:7673764C>T	p.E286K	chr17	7673764	7673764
Sample no. 43	Nonsense	SNV		g.chr17:7674939C>A	p.E198*	chr17	7674939	7674939
Sample no. 44	Missense	SNV	rs863224451	g.chr17:7673796C>A	p.C275F	chr17	7673796	7673796
Sample no. 47	Frame Shift Ins	INS		g.chr17:7674912_7674913insCAAATACTCCACACGCAAATTTCCTT	p.D207fs	chr17	7674912	7674913

	Sample no. 52	Splice Site	SNV		g.chr17:7674859C>T	p.E224E	chr17	7674859	7674859
	Sample no. 54	Nonsense	SNV	rs876660821	g.chr17:7675075A>C	p.H179Q	chr17	7675075	7675075
	Sample no. 55	Missense	SNV	rs121912656	g.chr17:7674229C>G	p.G245A	chr17	7674229	7674229
	Sample no. 8	Missense	SNV	rs28934574	g.chr17:7673776G>C	p.R282G	chr17	7673776	7673776
	Sample no. 9	Frame Shift Del	DEL		g.chr17:7676145delG	p.P75fs	chr17	7676145	7676145
	Sample no. 1	Nonsense	SNV	rs121913344	g.chr17:7673704G>A	p.R306*	chr17	7673704	7673704
	Sample no. 12	Splice Site	SNV		g.chr17:7674180C>A		chr17	7674180	7674180
	Sample no. 13	Missense	SNV	rs121912656	g.chr17:7674229C>T	p.G245D	chr17	7674229	7674229
XPC	Sample no. 25	Splice Site	DEL	rs750450365	g.chr3:14178466_14178468delCCT	p.Glu34del	chr3	14178466	14178468
	Sample no. 56	Missense	SNV		g.chr3:14158586C>T	p.E433K	chr3	14158586	14158586

Footnotes: SNV (single nucleotide variant), DEL (deletion), DNV (double nucleotide variant), INS (insertion), Chrom. (Chromosome)

Table S7. Results of significant differences in DNA repair gene expression stratified by the presence of somatic mutations. Differential expression analysis showed higher expression of several genes (*ERCC2*, *RECQL5*, *FAAP20*, *EXO1* and *PARP2*) in EOC patients with specific somatic mutations in *XPC* and *PRKDC*. Positive logFC (red color) indicates higher gene expression in EOC patients with specific potentially impactful somatic mutations compared to EOC patients without these mutations. List of the somatic mutations is summarized in the Table S6.

**Somatic mutation**	**Affected gene**	**logFC**	**Adj P-value**
XPC	*ERCC2*	2.01	0.003
	RECQL5	2.11	0.010
	FAAP20	1.82	0.040
PRKDC	*FAAP20*	1.90	0.032

Table S8. Associations of somatic mutations in DNA repair genes with *RBBP8* methylation. Positive logFC (red color) indicates a higher methylation profile in EOC patients bearing specific damaging somatic mutations compared to EOC patients bearing wild type genes.

Somatic mutation	Genome Change	Affected gene	logFC	Adj P-value
**Whole gene**				
BRCA1	g.chr17:43051062C>T; g.chr17:43076608delA	*RBBP8*	1.56	2.0×10^{-04}
RAD9A	g.chr11:67395816G>A; g.chr11:67392196G>A	*RBBP8*	1.33	0.013
**TSS200**				
BRCA1	g.chr17:43051062C>T; g.chr17:43076608delA	*RBBP8*	1.91	3.0×10^{-04}
RAD9A	g.chr11:67395816G>A; g.chr11:67392196G>A	*RBBP8*	1.90	4.0×10^{-04}
**TSS1500**				
BRCA1	g.chr17:43051062C>T; g.chr17:43076608delA	*RBBP8*	1.63	8.17×10^{-05}
RAD9A	g.chr11:67395816G>A; g.chr11:67392196G>A	*RBBP8*	1.40	5.0×10^{-04}
**Promoter**				
BRCA1	g.chr17:43051062C>T; g.chr17:43076608delA	*RBBP8*	1.69	1.0×10^{-04}
RAD9A	g.chr11:67395816G>A; g.chr11:67392196G>A	*RBBP8*	1.50	0.004

Table S9. Top correlated DNA repair gene expression (P < 0.01) with methylation profile

Gene	Correlation coefficient	P-value
Whole gene methylation vs. expression		
BRCA1	-0.379	0.003
FANCB	-0.342	0.007
MSH2	-0.355	0.005
TSS200 methylation vs. expression		
ERCC1	0.347	0.007
MUTYH	0.343	0.007
PER1	0.332	0.01
TSS1500 methylation vs. expression		
FANCG	-0.448	<0.001
MUTYH	0.417	0.001
Promoter methylation vs. expression		
ALKBH2	0.474	0.000
RAD9A	0.355	0.005

3 Supplementary Figures

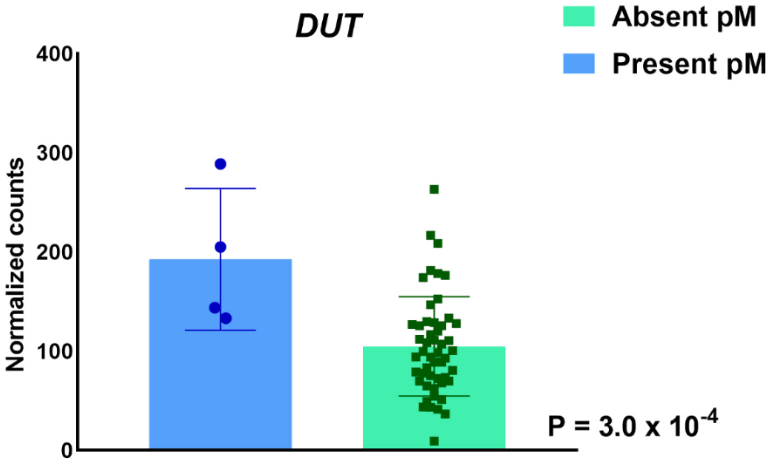

Figure S1. Differentially expressed gene *DUT* (deoxyuridine triphosphatase) stratified by the presence of peritoneal metastases (pM). Analysis of differential expression between patients with peritoneal metastases (N=4, blue color) or without (N=54, green color) showed significant downregulation of *DUT* gene in patients without peritoneal metastases.

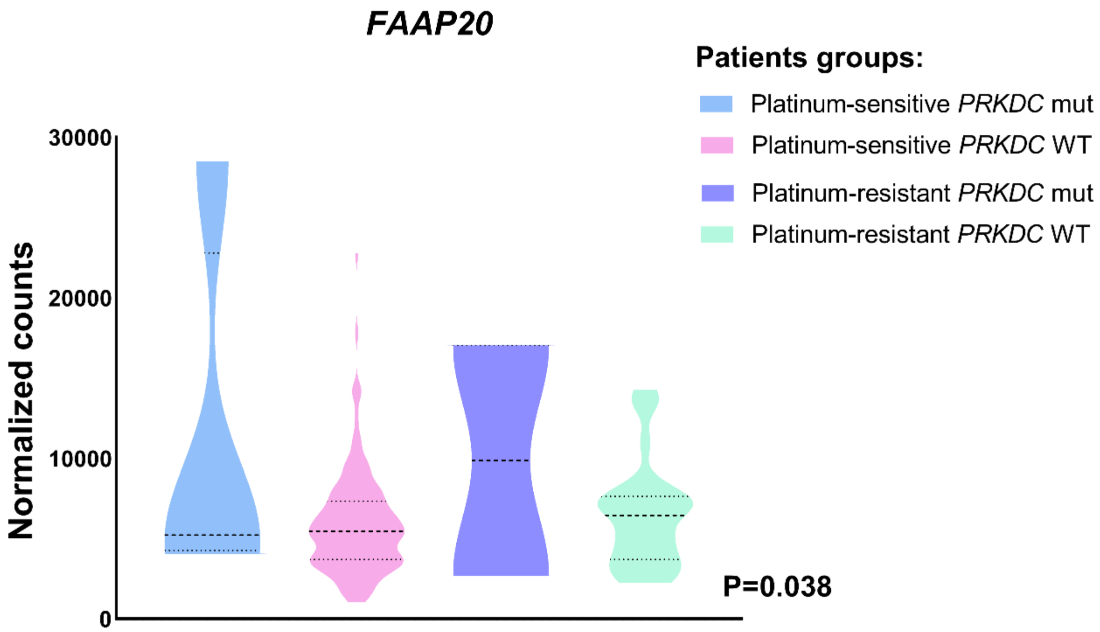

Figure S2. Differential *FAAP20* expression analysis stratified by the presence of somatic mutations in *PRKDC* and platinum-resistance in the TCGA dataset. We discovered a higher *FAAP20* expression in platinum-resistant patients harboring mutant *PRKDC* gene (N=2, purple color) compared to platinum-resistant ones harboring wild type *PRKDC* gene (N=29, green color) and platinum-sensitive patients harboring mutant *PRKDC* gene (N=4, blue color) or harboring wild type *PRKDC* gene (N=133, pink color).

A

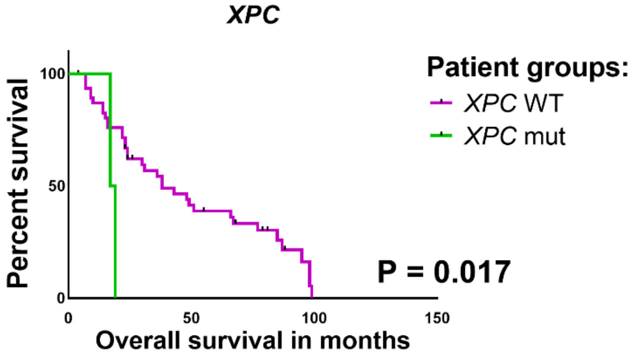

XPC	Total	Events	Censored	Median OS	95% CI
XPC mut	2	2	0	18	23.63 - 52.78
XPC WT	47	35	12	38	18.35 - 58.66

B

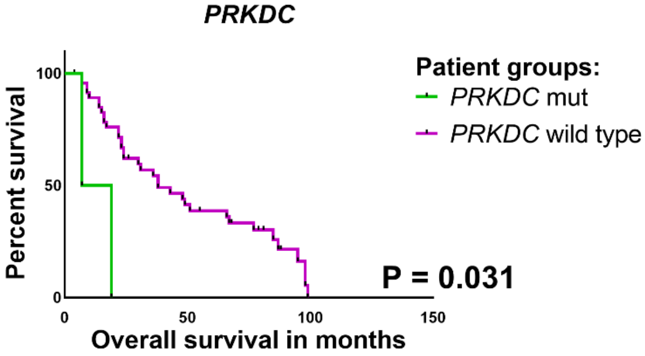

PRKDC	Total	Events	Censored	Median OS	95% CI
PRKDC mut	2	2	0	14	23.76 - 52.64
PRKDC WT	47	35	12	38	23.63 - 52.78

Figure S3. Kaplan-Meier plots of overall survival for (**A**) *XPC* and (**B**) *PRKDC* somatic mutations. Survival analysis showed significant association of *XPC* and *PRKDC* somatic mutations with overall survival of EOC patients.

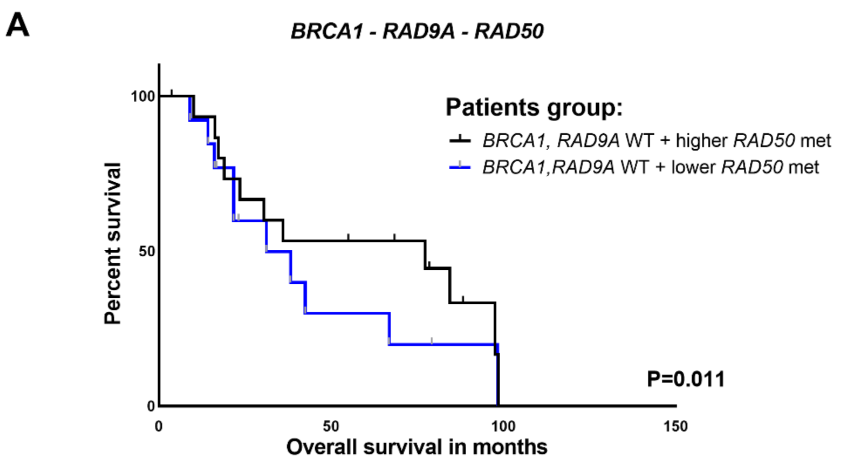

Group	Total	Events	Censored	Median OS	95% CI
BRCA1,RAD9A WT + lower *RAD50* met	17	11	5	18.9	9.66 - 28.14
BRCA1,RAD9A WT + higher *RAD50* met	14	9	4	77.2	61.22 - 93.19

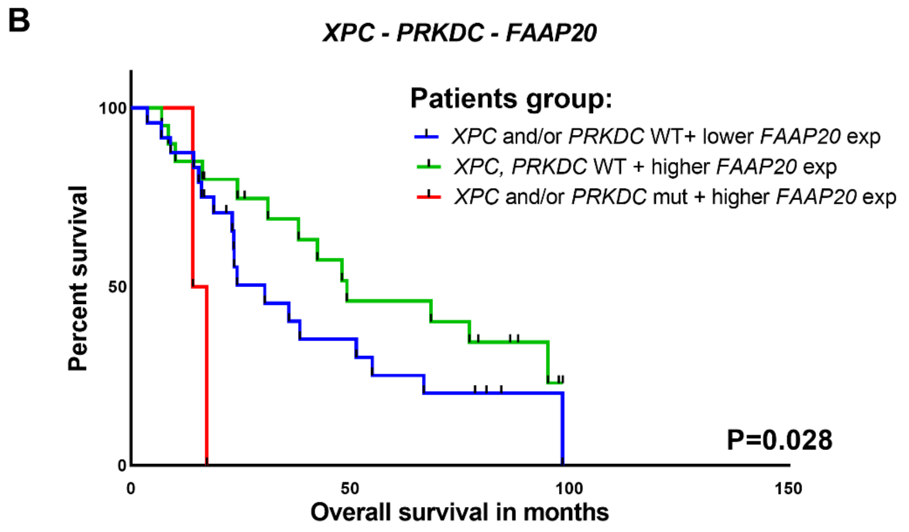

Group	Total	Events	Censored	Median OS	95% CI
XPC,PRKDC WT + higher *FAAP20* exp	22	13	7	49.5	14.44 - 36.36
XPC,PRKDC mut + higher *FAAP20* exp	3	2	0	15.7	0 - 4.82
XPC,PRKDC WT + lower *FAAP20* exp	25	18	6	30.5	15.18 - 21.82

Figure S4. Kaplan-Meier plots of overall survival for (**A**) *BRCA1-RAD9A-RAD50* and (**B**) *XPC-PRKDC-FAAP20* combinations.
